# Two odorant receptors regulate 1-octen-3-ol induced oviposition behavior in the oriental fruit fly

**DOI:** 10.1038/s42003-023-04551-5

**Published:** 2023-02-15

**Authors:** Li Xu, Hong-Bo Jiang, Jie-Ling Yu, Deng Pan, Yong Tao, Quan Lei, Yang Chen, Zhao Liu, Jin-Jun Wang

**Affiliations:** 1 Key Laboratory of Entomology and Pest Control Engineering, College of Plant Protection, Southwest University, Chongqing, 400716 China; 2 State Cultivation Base of Crop Stress Biology for Southern Mountainous Land, Academy of Agricultural Sciences, Southwest University, Chongqing, China

**Keywords:** Entomology, Molecular ecology

## Abstract

The oriental fruit fly *Bactrocera dorsalis* (Hendel) is a notorious pest of fruit crops. Gravid females locate suitable oviposition sites by detecting host plant volatiles. Here, we demonstrate that 1-octen-3-ol, a volatile from mango, guides the oviposition behavior of female flies. Two odorant receptors (BdorOR7a-6 and BdorOR13a) are identified as key receptors for 1-octen-3-ol perception by qPCR analysis, heterologous expression in *Xenopus laevis* oocytes and HEK 293 cells followed by in vitro binding assays, as well as CRISPR/Cas9 genome editing in *B. dorsalis*. Molecular docking and site-directed mutagenesis are used to determine major binding sites for 1-octen-3-ol. Our results demonstrate the potential of 1-octen-3-ol to attract gravid females and molecular mechanism of its perception in *B. dorsalis*. BdorOR7a-6 and BdorOR13a can therefore be used as molecular targets for the development of female attractants. Furthermore, our site-directed mutagenesis data will facilitate the chemical engineering of 1-octen-3-ol to generate more efficient attractants.

## Abstract (web summary)

Gravid female fruit flies show a strong preference to 1-octen-3-ol, a volatile compound from mango. Two odorant receptors are shown to regulate 1- octen-3-ol induced oviposition behaviour.

## Introduction

The selection of suitable oviposition sites by herbivorous insects usually reflects the ability of gravid females to detect volatiles released by preferred host plants. Examples include the fruit fly *Drosophila melanogaster*, which detects terpenes released by fermenting fruits^1,2^, the bean seed fly *Delia platura*, which detects 1-octen-3-ol and 3-octanone released by germinating seeds^3^, the silkworm moth *Bombyx mori*, which detects volatiles released by mulberry leaves^4^, and the parasitic wasp *Anastatus japonicas*, which detects β-caryophyllene, α-farnesene, and cis-3-hexen-ol released by host plants and insects^5^. The oriental fruit fly *Bactrocera dorsalis* (Hendel), one of the most destructive and invasive pests of fruit crops^6^, prefers to lay eggs on fully ripe mango fruit^7–10^. This is thought to reflect the attraction of gravid females to the volatiles. The chemical 1-octen-3-ol, a volatile from the mango fruit, was regarded as one of the possible orienteering cues for oviposition site selection in this fly^11,12^.

The olfactory system of insects plays an important role in the use of volatiles to guide oviposition behavior^13–15^. The odorant receptors (ORs) responsible for the perception of specific volatiles can be determined using a combination of exposure-based behavioral tests and loss-of-function mutations, with direct binding assays and site-directed mutagenesis as a strategy to identify specific binding sites^16^. Having characterized the volatiles and their receptors, reverse chemical ecology can be used to develop novel attractants that target key ORs more efficiently^13,17–19^. For example, DmelOR19a was found to be responsible for the perception of citrus terpenes that control oviposition behavior in *D. melanogaster*^2^. Similarly, HassOR31 was found to guide egg-laying behavior of *Helicoverpa assulta* in response to Z-3-hexenyl butyrate released by host plants^13^. Furthermore, we found that deleting the olfactory receptor co-expressed receptor (*BdorOrco*) gene using the CRISPR/Cas9 system abolished 1-octen-3-ol induced oviposition preference in female *B. dorsalis*^20^. The previous study has also shown directly that BdorOR13a binds to 1-octen-3-ol in vitro^16^, but no in vivo behavioral data has been presented thus far. Further analysis of the perception of 1-octen-3-ol in *B. dorsalis* has been hampered by an incomplete OR dataset due to the lack of a high-quality genome sequence.

To address these challenges, we comprehensively annotated the *B. dorsalis* OR family using a new, high-quality genome assembly. We identified two ORs that may be involved in the response to 1-octen-3-ol using expression analysis of ORs in virgin and mated females, and testing candidate ORs by heterologous expression systems followed by voltage clamp recording and calcium imaging assays. In addition, these two genes were knocked out using the CRISPR/Cas9 system. We used binding site analysis and site-directed mutagenesis to determine the binding mechanism. We also compared the effect of 1-octen-3-ol on gravid and virgin females. Our results indicate the molecular mechanism of 1-octen-3-ol perception in *B. dorsalis* and provide molecular targets for the development of more efficient female attractants based on the chemical engineering of 1-octen-3-ol.

## Results

### Behavioral assays

**Fig. 1:**
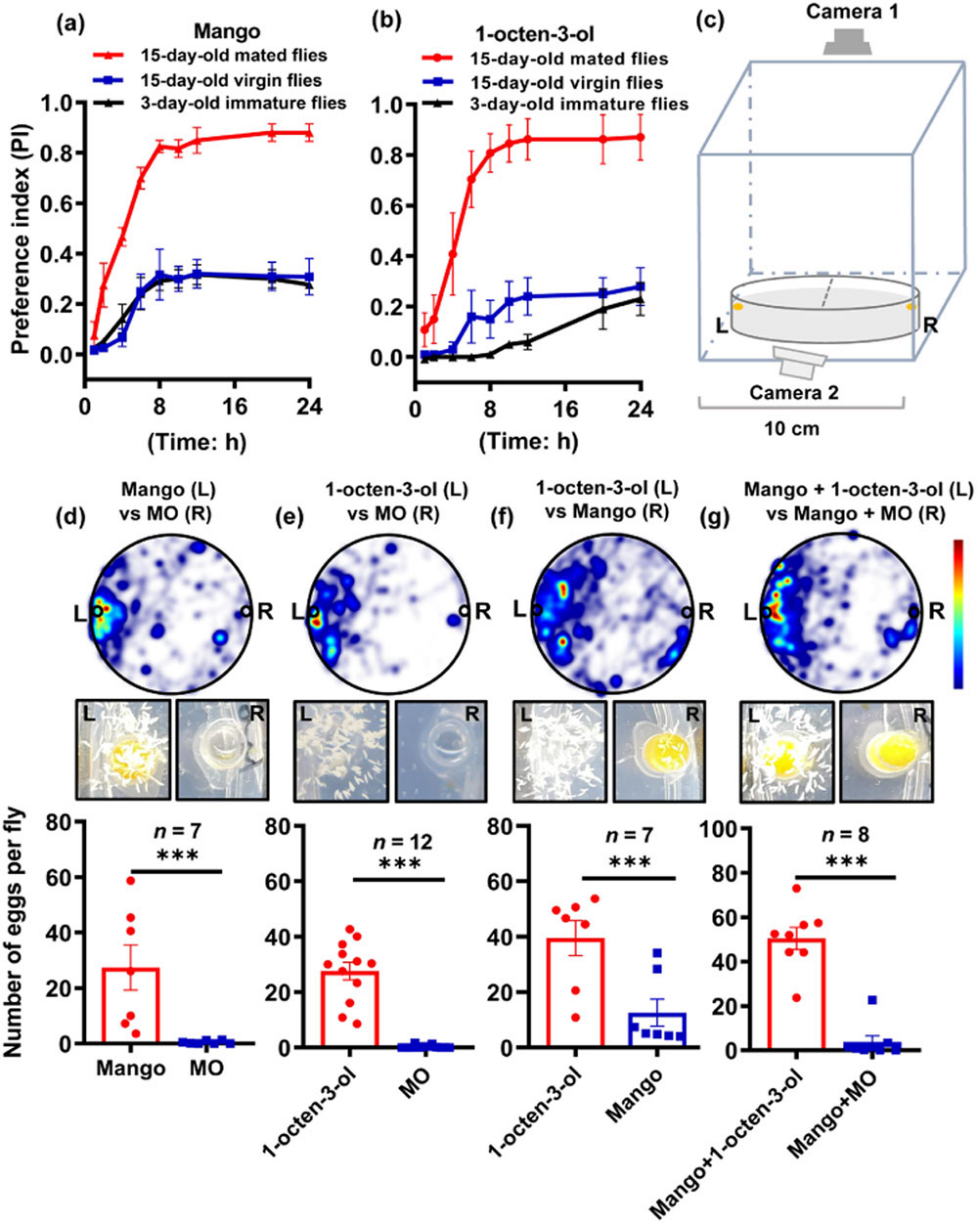
Olfactory preference and oviposition behavior in *B. dorsalis* induced by mango and 1-octen-3-ol. **a** The preference index of mango flesh for 3-day-old immature females, 15-day-old virgin females and 15-day-old mated females. **b** The preference index of 1-octen-3-ol for 3-day-old immature females, 15-day-old virgin females, and 15-day-old mated females. **c** Schematic diagram of the experimental setup for oviposition behavior assays. **d**–**g** Oviposition behavior induced by mango and/or 1-octen-3-ol. The heat map is based on fly tracks monitored using EnthoVision XT software. The color scheme represents the density of tracks and time spent of eight females, with red indicating the highest density. The images show the extent of oviposition after 24 h. The histograms show the average number of eggs laid by each female. Data are means ± SE of *n* ≥ 7 biological replicates. Statistical significance was determined using Student’s *t*-test (***p < 0.001). **d** Mango flesh vs MO. **e** 1-octen-3-ol vs MO. **f** Mango flesh vs 1-octen-3-ol. **g** Mango flesh + 1-octen-3-ol vs mango flesh + MO.

We compared the ability of fully ripe mango flesh and 1-octen-3-ol to attract virgin and mated female *B. dorsalis* using trap lures. The fully ripe mango flesh (Fig. 1a) and the 10% (v/v) 1-octen-3-ol (diluted by mineral oil (MO)) (Fig. 1b) were both significantly more attractive to 15-day-old mated females than 15-day-old virgin females and 3-day-old immature females, but there was no significant difference between the virgin and immature females. Furthermore, the preference index (the number of flies in odorant trap minus the number of flies in MO trap and then divided by total introduced flies) of gravid females to both ripe mango and 1-octen-3-ol became higher over time. Almost all of the females made their choice after ~8 h and remained relatively stable thereafter.

We studied the effect of ripe mango and 1-octen-3-ol on oviposition behavior using the experimental approach shown in Fig. 1c. Briefly, we placed ~60 mg of mango flesh or 20 µL of 1-octen-3-ol solution on one side of a Petri dish (point L), and 20 µL MO on the opposing side (point R), then tracked the flies. When mango flesh was placed at point L and MO at point R, the mean number of eggs laid by each female was 27 ± 8 (*n* = 7) at point L and almost zero at point R (*p* < 0.001, Student’s *t* test), and the fly tracks were mainly concentrated around the mango flesh (Fig. 1d). Similar results were observed when 1-octen-3-ol was applied at point L and MO at point R (Fig. 1e). When mango flesh was tested directly against 1-octen-3-ol, the flies laid significantly more eggs in the 1-octen-3-ol region (Fig. 1f). Furthermore, gravid females showed a preference for mango flesh supplemented with 1-octen-3-ol compared to mango flesh supplemented with MO, and the number of eggs was higher compared to mango flesh or 1-octen-3-ol alone (Fig. 1g). The tracks of flies concentrated around the preferred oviposition site in all experiments. These results indicated that 1-octen-3-ol could be suitable as a lure for the control of *B. dorsalis* females. The oviposition behavior is shown in more detail in Supplementary Movies 1–4.

### Annotation of *B. dorsalis* OR genes

We identified 74 *B. dorsalis* OR genes by using BLASTX to screen *B. dorsalis* genomic contigs against the amino acid sequences and Pfam domains of known *D. melanogaster* ORs with an identity cut-off of 30% (Supplementary Table 1, Supplementary Data 1). The contigs were derived from a high-quality final *B. dorsalis* genome assembly that has been submitted to CNGBdb (accession number CNP0003192). We also included several *B. dorsalis* tissue transcriptomes including antenna (GenBank SRR9026238), maxillary palps and proboscis (accession number CNP0003333), and other tissues (accession number CNP0003334). We used a BLASTN search to predict the full-length coding sequences of ORs and determined their sequences by RT-PCR. A homology search based on the amino acid sequences of *D. melanogaster* ORs revealed multiple homologous genes for several ORs including *BdorOR7a*, *BdorOR33b*, *BdorOR59a*, *BdorOR63a, BdorOR67d*, and *BdorOR94a*, whereas the corresponding *Drosophila* ORs have only one gene (Supplementary Fig. 1).

### Expression profiles of *B. dorsalis* OR genes

The expression profiles of *B. dorsalis* OR genes were investigated by testing seven tissues in different *B. dorsalis* body segments by qPCR (Supplementary Fig. 2). Most genes were strongly expressed in tissues rich in sensilla, such as maxillary palps, head cuticles, and especially the antennae. Several genes were also expressed in the proboscis, including *BdorOR7a-4*, *BdorOR19a*, *BdorOR24a*, *BdorOR47b,* and *BdorOR94a-2*. We compared the gene expression profiles in 15-day-old virgin and mated females, revealing 20 genes that were significantly upregulated and five genes that were significantly downregulated after mating (Supplementary Fig. 3). Given that the gravid females were more sensitive to 1-octen-3-ol, we considered the 20 upregulated OR genes as candidates for the analysis of 1-octen-3-ol binding affinity.

### Affinity of candidate ORs for 1-octen-3-ol in vitro

**Fig. 2:**
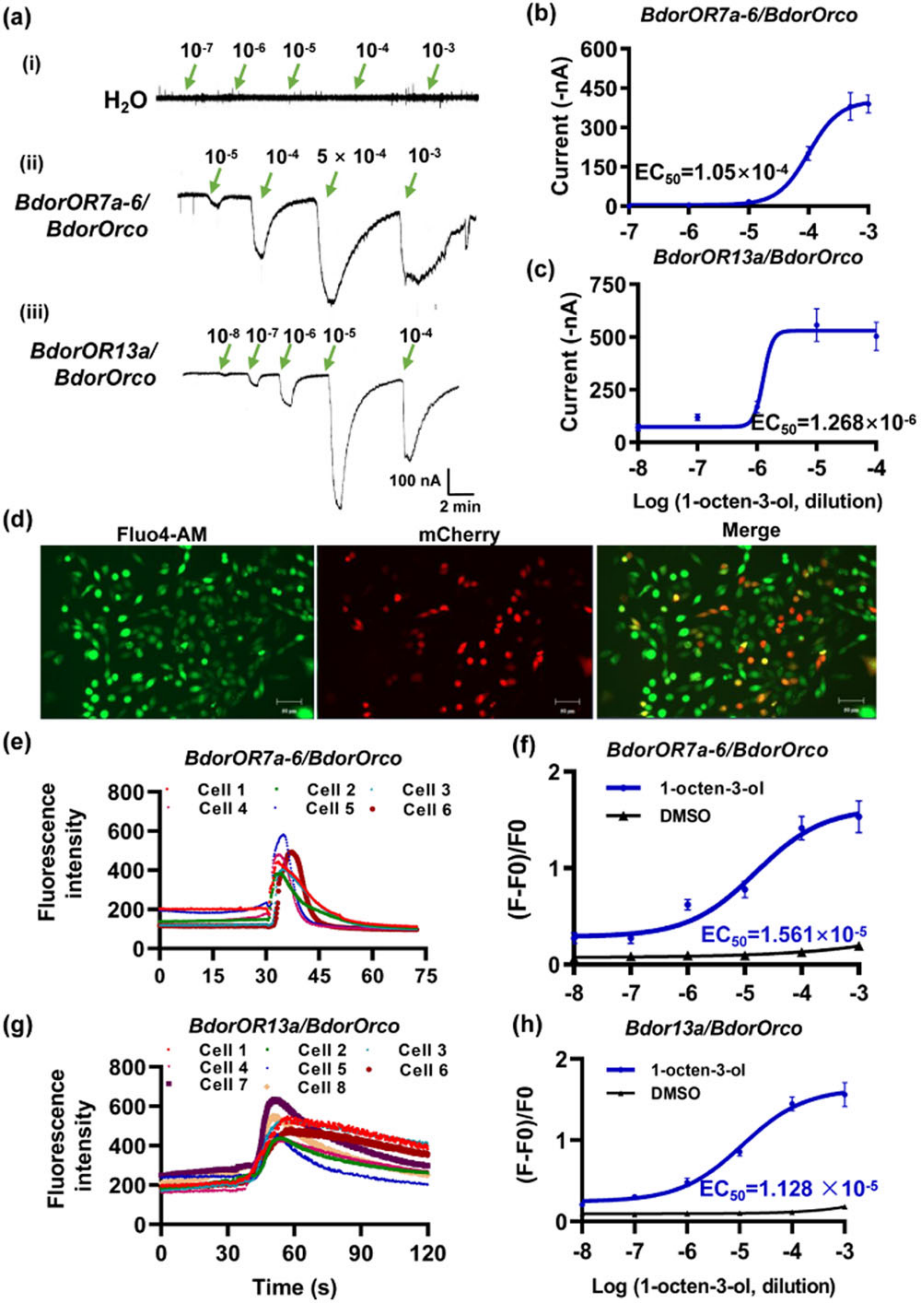
Responses of *BdorOR7a-6/BdorOrco* and *BdorOR13a/BdorOrco* to 1-octen-3-ol. **a**–**c** Responses to 1-octen-3-ol of *Xenopus* oocytes co-expressing *BdorOR7a-6*/*BdorOrco* or *BdorOR13a/BdorOrco*. **a** *Xenopus* oocytes were injected with the appropriate constructs and stimulated with 1-octen-3-ol at different concentrations. The oocytes were injected with (i) water as a control, (ii) cRNA of *BdorOR7a-6/BdorOrco*, (iii) cRNA of *BdorOR13a/BdorOrco*. **b** Dose-response curve of BdorOR7a-6 to 1-octen-3-ol. EC_50_ = 1.05 × 10^−4^ M. **c** Dose-response curve of BdorOR13a to 1-octen-3-ol. EC_50_ = 1.268 × 10^−6^ M. Symbols show the current responses from the BdorOR/BdorOrco complex presented as means ± SE (*n* = 8). The dose–response curves were fitted using GraphPad 8.0. **d**–**h** Responses to 1-octen-3-ol of HEK 293 cells co-expressing *BdorOR7a-6*/*BdorOrco* or *BdorOR13a/BdorOrco*. **d** Fluorescence images of HEK 293 cells incubated with Fluo4-AM and transfected with the candidate OR genes. Fluo4-AM was used to indicate the fluorescence change (ΔF) over time, and mCherry was used as the reporter to observe the successfully transfected cells. The proportional scale is 50 μm. **e** Change in fluorescence intensity of cells expressing *BdorOR7a-6*/*BdorOrco* following stimulation with 10^−4 ^M of 1-octen-3-ol. **f** Dose–response curve of BdorOR7a-6 to 1-octen-3-ol. EC_50_ = 1.561 × 10^−5^ M. **g** Change of fluorescence intensity of cells expressing *BdorOR13a/BdorOrco* following stimulation with 10^−4 ^M of 1-octen-3-ol. **h** Dose–response curve of BdorOR13a to 1-octen-3-ol. EC_50_ = 1.128 × 10^−5^ M.

To determine which *B. dorsalis* ORs are primarily responsible for the perception of 1-octen-3-ol, we expressed all 20 candidates in *Xenopus laevis* oocytes along with the co-receptor gene *BdorOrco*, and used a two-electrode voltage clamping system to record the response to 1-octen-3-ol. Control oocytes injected with water did not generate detectable currents when challenged with either DMSO or 1-octen-3-ol (Fig. 2a). Among the 20 candidate ORs, only oocytes expressing BdorOR7a-6/BdorOrco or BdorOR13a/BdorOrco were clearly activated by 1-octen-3-ol, and in both cases we observed dose-dependent currents (Fig. 2b, c). The half-maximal effective concentration (EC_50_ value) of 1-octen-3-ol, which induces a response halfway between the baseline and maximum, was 105 μM for BdorOR7a-6/BdorOrco and 1.268 μM for BdorOR13a/BdorOrco. BdorOR13a therefore appears to have a much greater affinity than BdorOR7a-6 for 1-octen-3-ol.

To confirm the two-electrode voltage clamping data, we expressed the ORs in HEK 293 cells and detected ability of the ORs to bind 1-octen-3-ol by calcium imaging. Cells incubated with Fluo-4 AM were green when excited at 488 nm, whereas cells successfully transfected with the OR constructs and the visual marker mCherry were red when excited at 555 nm (Fig. 2d). Only cells transfected with BdorOR7a-6/BdorOrco or BdorOR13a/BdorOrco showed a significant change in green fluorescence intensity following stimulation with 10^−4^ M 1-octen-3-ol (Fig. 2e, g), and the concentration–response curves revealed EC_50_ values of 15.6 μM for BdorOR7a-6/BdorOrco and 11.3 μM for BdorOR13a/BdorOrco (Fig. 2f, h).

### Genome editing of *BdorOR7a-6* and *BdorOR13a*

**Fig. 3:**
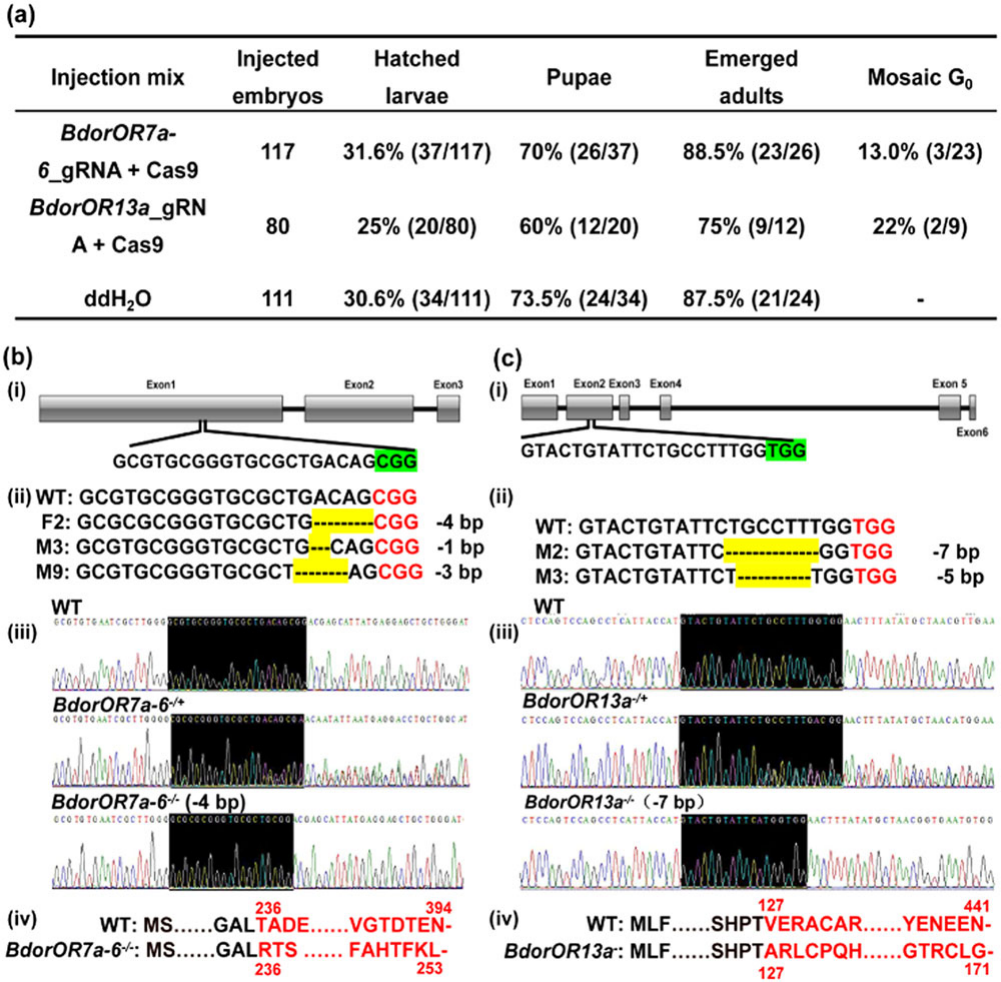
CRISPR/Cas9 mutagenesis of *BdorOR7a-6* and Bdor*OR13a*. **a** Rates of survival and mutagenesis after microinjection. **b** The target region of *BdorOR7a-6*. (i) Gene structure of *BdorOR7a-6* based on the genome sequence. Exons are shown as gray boxes and introns as lines. The gRNA target in the first exon contains a 20-nt guide sequence and the adjacent CGG highlighted in green is the protospacer adjacent motif (PAM). (ii) The genotypes of G_0_ mutants were determined by TA cloning and Sanger sequencing (deletions are shown as dashes highlighted in yellow). The identifier assigned to the mosaic G_0_ flies is shown in the front of each sequence, and the number of the deleted nucleotides is shown after each sequence. (iii) Sanger sequencing traces for WT, *BdorOR7a-6*^*−/+*^, and *BdorOR7a-6*^*−/−*^ flies. The black region is the gRNA target site. (iv) Predicted protein sequences of the BdorOR7a-6 WT and mutant (−4 bp) alleles. **c** The target region of *BdorOR13a*, with (i–iv) indicating the same details as provided for above the *BdorOR7a-6* mutants.

We designed gRNAs against the *BdorOR7a-6* and *BdorOR13a* genes and injected them into freshly laid *B. dorsalis* eggs along with purified Cas9 protein to generate knockout mutations. The *BdorOR7a-6* and *BdorOR13a* target sites were located in the first and second exons, respectively (Fig. 3b, c). We obtained 26 adults in the *BdorOR7a-6* group and 12 in the *BdorOR13a* group. Mosaic G_0_ individuals were identified based on the analysis of polymorphisms, and the G_0_ mutation efficiency was found to be 13% and 22%, respectively (Fig. 3a). TA cloning and Sanger sequencing revealed three genotypes among the G_0_ *BdorOR7a-6*^*–/+*^ mutants and two among the G_0_ *BdorOR13a*^*–/+*^ mutants. The mutants were individually crossed with wild-type (WT) flies, and the G_1_ offspring were backcrossed to WT flies for at least 10 generations to exclude potential off-target mutations. Finally, we obtained *BdorOR7a-6*^*−/−*^ flies with a homozygous 4-bp deletion and *BdorOR13a*^*−/−*^ flies with a homozygous 7-bp deletion (Fig. 3b, c). The BdorOR7a-6 amino acid sequence in the mutants was altered from position 236 and created a stop codon at position 254 (compared to the 394-residue WT protein). The BdorOR13a amino acid sequence in the mutants was altered from position 127 and created a stop codon at position 172 (compared to the 441-residue WT protein).

### Phenotypic analysis of the *BdorOR7a-6*^*−/−*^ and *BdorOR13a*^*−/−*^ mutants

**Fig. 4:**
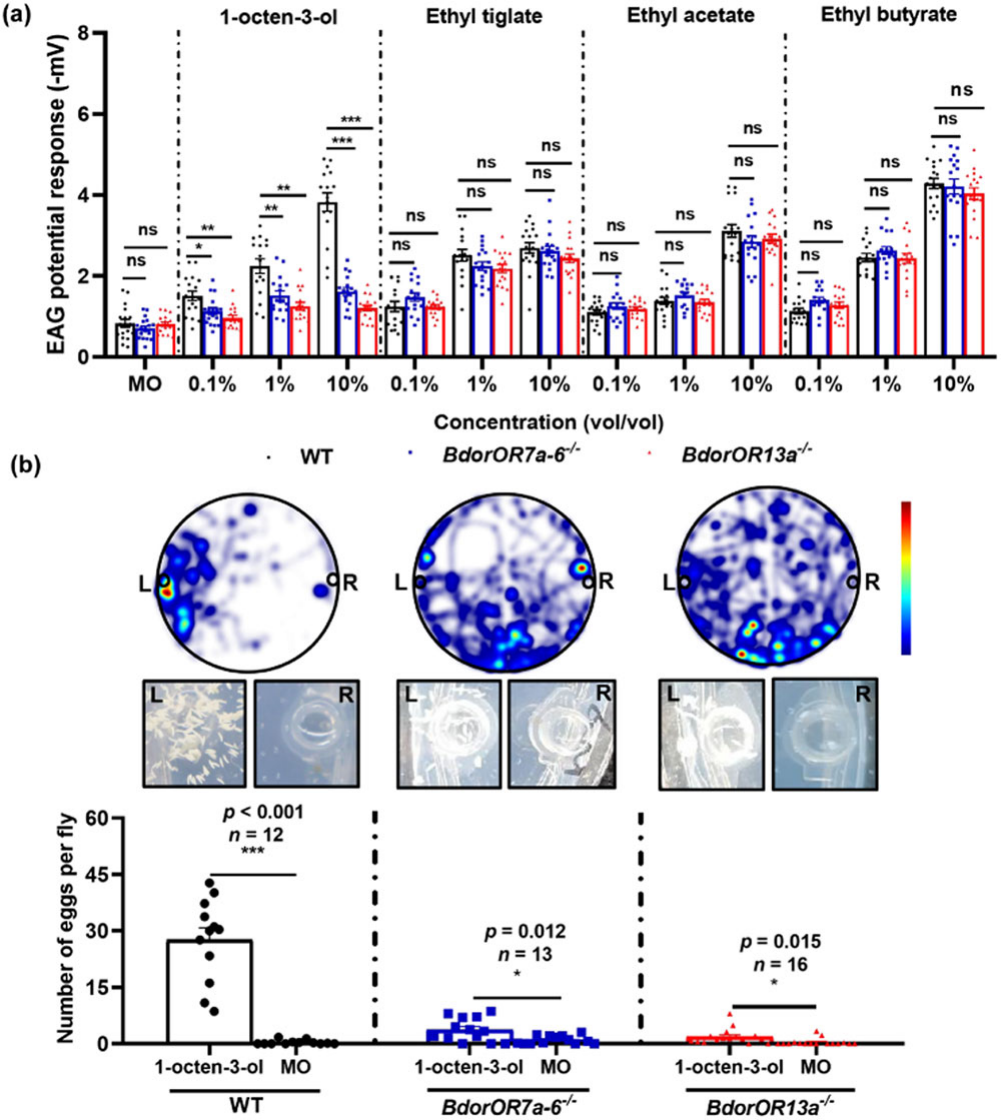
The phenotypes of *BdorOR7a-6*^*−/−*^ and *BdorOR13a*^*−/−*^ mutants. **a** EAG responses of WT, *BdorOR7a-6*^*−/−*^ and *BdorOR13a*^*−/−*^ mutants to different concentrations of 1-octen-3-ol and other volatiles. Data are means ± SE (*n* = 15–20). Statistical significance was determined using Student’ s *t*-test (*p < 0.05, **p < 0.01, ***p < 0.001). **b** Oviposition behavior induced by 1-octen-3-ol in WT and mutant females. The heat map shows the density of tracks and time spent of eight females, with red indicating the highest density. The images show the extent of oviposition after 24 h, and the histogram shows the average number of eggs laid by each fly. Data are means ± SE (*n* ≥ 8 biological replicates). Statistical significance was determined using Student’s *t*-test (***p < 0.001).

We compared the electrophysiological responses of mutant and WT flies to 1-octen-3-ol and other volatiles by electroantennography (EAG). The average EAG response of WT flies increased gradually with the concentration of 1-octen-3-ol (Fig. 4a), reaching a peak of 3.829 ± 0.33 mV at 10% (v/v) 1-octen-3-ol. The average EAG responses of the mutants were significantly lower at 0.1%, 1%, and 10% (v/v) 1-octen-3-ol compared to the WT flies. Furthermore, the EAG responses of the *BdorOR13a*^*−/−*^ mutants were lower than those of the *BdorOR7a-6*^*−/−*^ mutants at 1% and 10% (v/v) 1-octen-3-ol. However, the EAG response of *BdorOR7a-6*^*−/−*^ and *BdorOR13a*^*−/−*^ mutants to other three volatiles (ethyl tiglate, ethyl acetate, and ethyl butyrate) showed no significant difference compared with WT flies.

Oviposition bioassays were carried out to compare the efficiency of 1-octen-3-ol as an attractant to WT and mutant females, using the same experimental setup as described above (Fig. 1c). The mean number of eggs laid by each WT female at the 1-octen-3-ol site was 28 ± 3 (*n* = 12), compared to almost 0 (*n* = 12) eggs at the site treated with MO (*p* < 0.001, Student’s *t* test, Fig. 4b). However, the mean number of eggs laid at the 1-octen-3-ol site by the mutant flies was significantly lower: 4 ± 1 (*n* = 13, *p* < 0.001, Student’s *t* test, Fig. 4b) for *BdorOR7a-6*^*−/−*^ mutants and 2 ± 1 (*n* = 16, *p* < 0.001, Student’s *t* test, Fig. 4b) for *BdorOR13a*^*−/−*^ mutants. There was no significant difference between WT flies and mutant flies at the MO site. Furthermore, the tracks of the *BdorOR7a-6*^*−/−*^ and *BdorOR13a*^*−/−*^ mutants were disordered, covering the entire Petri dish, in contrast to the WT tracks concentrated around the attractant. The behaviors of the *BdorOR7a-6*^*−/−*^ and *BdorOR13a*^*−/−*^ mutants are shown in Supplementary Movies 5–6.

### Molecular docking and site-directed mutagenesis

**Fig. 5:**
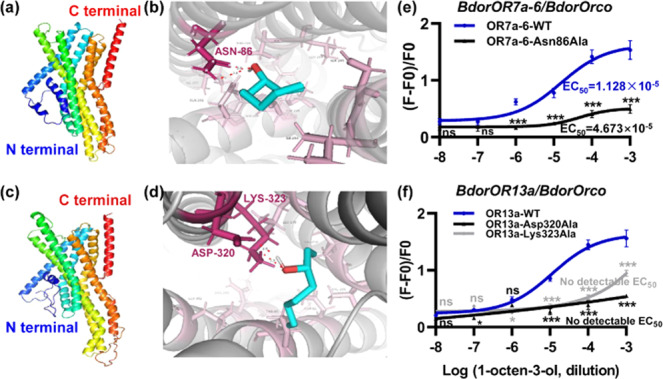
Molecular docking of BdorOR7a-6 and BdorOR13a to 1-octen-3-ol, and site-directed mutagenesis to confirm the essential residues. **a** Structure of BdorOR7a-6 predicted using AlphaFold 2.0. **b** Residue of BdorOR7a-6 required for binding to 1-octen-3-ol. **c** Structure of BdorOR13a predicted using AlphaFold 2.0. **d** Residues of BdorOR13a required for binding to 1-octen-3-ol. **e** Response of BdorOR7a-6 Asn86Ala mutant to 1-octen-3-ol based on calcium imaging. **f** Responses of BdorOR13a Asp320Ala and Lys323Ala mutants to 1-octen-3-ol based on calcium imaging.

We used AlphaFold 2.0 to predict the structures of BdorOR7a-6 and BdorOR13a (Fig. 5a, c). The accuracy of the prediction was assessed using a PROCHECK Ramachandran plot, revealing that 96.7% of the BdorOR7a-6 residues were placed in favored regions (A, B, L) and no residues were placed in disallowed regions (Supplementary Fig. 4a). Similarly, 95.3% of the BdorOR13a residues were placed in favored regions and no residues were placed in disallowed regions (Supplementary Fig. 4b). Molecular docking showed that BdorOR7a-6 and BdorOR13a feature an elongated pocket-like cavity that binds 1-octen-3-ol. Residue Asn86 of BdorOR7a-6 (Fig. 5b), and residues Asp320 and Lys323 of BdorOR13a (Fig. 5d), were predicted to form hydrogen bonds with 1-octen-3-ol. The veracity of the three binding sites was determined by site-directed mutagenesis, in which each site was individually replaced with alanine. The three mutant sequences were transfected into HEK 293 cells along with the *BdorOrco* construct, followed by calcium imaging as described above. Mutation Asn86Ala significantly reduced the binding affinity of BdorOR7a-6 for 1-octen-3-ol, increasing the EC_50_ value to 46.7 μM. Mutations Lys323Ala and Asp320Ala abolished the ability of BdorOR13a to bind 1-octen-3-ol, in each case making it impossible to record an EC_50_ value (Fig. 5e, f). In addition, the molecular docking results showed that the three mutations could not form hydrogen bonds with 1-octen-3-ol (Supplementary Fig. 5–6).

## Discussion

The main component of the widely used commercial attractant for *B. dorsalis* is methyl eugenol, which strongly attracts males but not females^21^. In the field, the attraction of males has no beneficial effect after mating because this does not prevent oviposition by gravid females and the subsequent damage caused by larvae. The successful control of *B. dorsalis* therefore requires either mating disruption or a female attractant. Gravid females prefer to lay eggs in ripe mango fruits^7–10^, and 1-octen-3-ol was proposed as one of the olfactory cues that may guide this oviposition behavior^11,12^. We found that 1-octen-3-ol is more attractive to mated females than virgins, making it an ideal candidate for the development of a female attractant. Interestingly, when mango and 1-octen-3-ol were offered as alternative choices, 1-octen-3-ol was the preferred choice and also induced the gravid females to lay more eggs. This indicates that 1-octen-3-ol not only attracts females, but also stimulates oviposition activity.

*BdorOrco*^*−/−*^ mutants do not respond to 1-octen-3-ol in EAG experiments and show a significant change in oviposition behavior in our previous study^20^. Given that Orco is strictly required for olfactory cue perception in insects, oviposition behavior guided by 1-octen-3-ol must be mediated by ORs. Although several attempts have been made to identify the ORs for 1-octen-3-ol, the molecular basis of its perception has remained largely unknown thus far due to the lack of a high-quality genome sequence^16^. We therefore annotated *B. dorsalis* OR genes using a high-quality genome assembly and several transcriptomic databases. We found 74 *B. dorsalis* OR candidate genes, the most comprehensive repository thus far, 64 of which we cloned by RT-PCR and confirmed by Sanger sequencing. These data provide a solid foundation for the functional characterization of more ORs in the future. Phylogenetic analysis of ORs from *B. dorsalis* and *D. melanogaster* revealed some direct orthologs but other cases of gene duplication and divergence, as previously reported^16^, reflecting adaptions to environmental odorants such as plant volatiles. It would be intriguing to determine whether these homologous ORs are involved in the perception of specific odorants or sets of analogous odorants. The *B. dorsalis* OR genes are mainly expressed in olfactory organs, as previously reported for *D. melanogaster*^22^. Although sexually dimorphic responses to volatiles have been reported in *B. dorsalis*^21,23,24^, we observed no significant transcriptomic differences between males and females, in agreement with previous results^16^.

Mating status is a key switch for insects, allowing them to enter the ready-to-oviposit state that triggers oviposition behavior^25^. For example, mated females of the Mediterranean fruit fly *Ceratitis capitata* switch preference from male pheromones to host fruit odorants^26^. The mating status of females is predicted to influence their perception of volatiles along with their new behavioral state and physiological needs^27^, and accordingly we found that 1-octen-3-ol was more attractive to mated than virgin females in the present study. We found 20 *B. dorsalis* OR genes that were upregulated in mated females, and considered them as candidate ORs for the perception of 1-octen-3-ol, but only BdorOR7a-6 and BdorOR13a showed a significant response to 1-octen-3-ol. Orthologs of BdorOR13a are similarly responsive to 1-octen-3-ol in *D. melanogaster*^28^, *Anopheles gambiae*^29^, and *Spodoptera frugiperda*^30^.

Heterologous expression systems, including *X. laevis* oocytes, *S. frugiperda* (Sf9) cells, HEK 293 cells, and the *Drosophila* “empty neuron” system, have been used to identify ligands for orphan ORs^31^. The voltage clamp recording showed that BdorOR7a-6 had a significantly larger EC_50_ value than BdorOR13a, whereas calcium imaging showed no significant difference between the receptors. These incongruent results may reflect the inherent characteristics of each heterologous system and/or differences in the measurement techniques^32^. In addition, our voltage clamp recording results in other study showed BdorOR7a-6 could respond to the other two volatiles, indicating BdorOR7a-6 was broadly tuned to host volatiles compared to BdorOR13a.

The oviposition behavior induced by 1-octen-3-ol was significantly altered in the *BdorOR7a-6*^*−/−*^ and *BdorOR13a*^*−/−*^ mutants. Gravid mutant females laid far fewer eggs than WT flies following stimulation with 1-octen-3-ol, and the reduced olfactory sensitivity to 1-octen-3-ol also disrupted the tracks of the mutant flies. Videos S1–S4 show that WT females quickly located the 1-octen-3-ol and then laid eggs around it, whereas the mutants were in many cases unable to locate the 1-octen-3-ol. However, the females still laid significantly more eggs on the 1-octen-3-ol treated side than MO. This makes sense given that we did not perform the double knockouts, so the other OR could partially rescue egg laying. BdorOR13a and BdorOR7a-6 therefore appear to be tuned to 1-octen-3-ol, and this regulates oviposition behavior. This is consistent with findings in the mosquito *Culex quinquefasciatus*, where receptors CquiOR37 and CquiOR99 are narrowly tuned to two oviposition attractants (4-methylphenol and 4-ethylphenol), but this preference is lost in the mutants *CquiOR37*^*−/−*^ and *CquiOR99*^*−/−* ^^33^. OR-dependent olfactory responses are therefore necessary for odorant-directed oviposition behavior. The *BdorOR7a-6*^*−/−*^ and *BdorOR13a*^*−/−*^ mutants also showed a significantly lower EAG response to 1-octen-3-ol than WT flies, with *BdorOR13a*^*−/−*^ mutants showing the greatest deficiency. The EC_50_ value of BdorOR13a (1.27 µM) was also much lower than that of BdorOR7a-6 (105 µM) in the voltage clamp assay. These data suggest that BdorOR13a is the primary OR for the perception of 1-octen-3-ol although each receptor can partially compensate for the knockout of the other.

Site-directed mutagenesis can be used to explore the binding properties of insect ORs and odorants^34–36^. In this study, the Asp320Ala and Lys323Ala mutants of BdorOR13a were unable to bind 1-octen-3-ol, suggesting the mutation altered the conformation of the binding pocket. The Asn86Ala mutant of BdorOR7a-6 retained its ability to bind 1-octen-3-ol but the binding affinity was much lower, indicating that the compact structure of the binding site may have relaxed^37^. Similar results were reported in *Ostrinia furnacalis*^38^, *D. melanogaster*^39^, and *A. gambiae*^40^. Our results show that the conformation of ORs is affected by the replacement of single amino acid, and that the Asp320 and Lys323 residues of BdorOR13a are absolute requirements for 1-octen-3-ol binding. The further analysis of OR structures may provide more insight into the conformational changes involved in odorant binding, facilitating the screening of odorants that attract *B. dorsalis* more efficiently by binding to ORs with greater affinity.

In conclusion, we found that 1-octen-3-ol is a strong attractant for gravid *B. dorsalis* females and also regulates their oviposition behavior. We completed the genome-wide annotation of *B. dorsalis* ORs and used in vitro binding assays and genome editing followed by behavioral tests to show that two receptors (BdorOR13a and BdorOR7a-6) are tuned to 1-octen-3-ol. Our results not only show the potential of 1-octen-3-ol for the attraction of gravid *B. dorsalis* females, but also reveal the molecular basis of its perception. This may facilitate the development of more potent female attractants to reduce the impact of *B. dorsalis* on fruit crops.

## Methods

### Insect rearing

WT *B. dorsalis* were collected from Haikou, Hainan province, China, in 2008. They were maintained at the Key Laboratory of Entomology and Pest Control Engineering in Chongqing at 27 ± 1 °C, 70 ± 5% relative humidity, with a 14-h photoperiod. Adult flies were reared on an artificial diet containing honey, sugar, yeast powder, and vitamin C. Newly hatched larvae were transferred to an artificial diet containing corn and wheat germ flour, yeast powder, agar, sugar, sorbic acid, linoleic acid, and filter paper.

### Behavioral assays

Double trap lure assays were set up to compare the olfactory preferences of gravid and virgin females in a 20 × 20 × 20 cm transparent cage with evenly distributed holes (diameter = 1.5 mm) on the side walls. The traps were refitted from inverted 50-mL centrifuge tubes and were placed along the diagonal of the cage. The top of each trap was pierced with a 1-mL pipette tip, which was shortened to ensure flies could access the trap from the pipette. For the olfactory preference assay with mango, one trap was loaded with 60 mg mango flesh and the other trap with 20 μL MO in the cap of a 200-μL PCR tube. For the olfactory preference assay with 1-octen-3-ol (≥98%, sigma, USA), one trap was loaded with 20 μL 10% (v/v) 1-octen-3-ol diluted in MO, and the other with 20 μL MO. A cotton ball soaked in water was placed at the center of the cage to provide water for the flies. Groups of 30 female flies were introduced into the cage for each experiment, and each experiment was repeated to provide eight biological replicates. All experiments commenced at 10 am to ensure circadian consistency. The number of flies in each trap was counted every 2 h for 24 h. We compared the preferences of 3-day-old immature females, 15-day-old virgin females, and 15-day-old mated females. The olfactory preference index was calculated using the following formula^41^: (number of flies in mango/odorant trap – number of flies in control trap)/total number of flies.

Oviposition behavior was monitored in a 10 × 10 × 10 cm transparent cage with evenly distributed holes on the side walls as above. A 9-cm Petri dish filled with 1% agar was served as an oviposition substrate, and the mango flesh, 10% (v/v) 1-octen-3-ol or MO were added at opposite edges of the dish. We tested the preference of flies for different substrates: (1) ~60 mg of mango flesh on one edge and 20 μL of MO on the other; (2) 20 μL of 1-octen-3-ol on one edge and 20 μL of MO on the other; (3) ~60 mg mango flesh on one edge and 20 μL of 1-octen-3-ol on the other; and (4) ~60 mg mango flesh plus 20 μL 1-octen-3-ol on one side and ~60 mg of mango flesh plus 20 μL MO on the other. The agar disc was covered in a pierced plastic wrap to mimic fruit skin, encouraging flies extend their ovipositor into the plastic wrap to lay eggs. The agar disc was placed at the center of the cage, and we introduced eight 15-day-old gravid females. Two Sony FDR-AX40 cameras recorded the behavior of the flies for 24 h, one fixed above the cage to record the tracks and the other placed in front of the cage to record the oviposition behavior. Based on the results from double traps luring assays, a 3 h duration (6–9 h) of the videos was selected to analyze the tracks and spent time of all flies in observed area (the surface of Petri dish). The videos were analyzed using EthoVision XT v16 (Noldus Information Technology) to determine the total time of all flies spent on each side in seconds and the total distance of movement in centimeters, and the tracks were visualized in the form of heat maps^17^. The number of eggs laid by the eight flies in each experiment was counted under a CNOPTEC stereomicroscope, and each experimental group comprised 7–16 replicates.

### Annotation of *B. dorsalis* OR genes

*D. melanogaster* amino acid sequences downloaded from the National Center for Biotechnology Information (https://www.ncbi.nlm.nih.gov/) were used as BLASTP queries against the *B. dorsalis* amino acid database with an identity cut-off of 30%. The candidate OR genes were compared with deep transcriptome data from *B. dorsalis* antennae^42^, maxillary palps and proboscis, and other tissues.

### Cloning of candidate *B. dorsalis* OR genes

High-fidelity PrimerSTAR Max DNA polymerase (TaKaRa, Dalian, China) was used to amplify the full open reading frame of *BdorOR* genes by nested PCR using primers (Supplementary Table 2) designed according to *B. dorsalis* genome data. Each 25-μL reaction comprised 12.5 μL 2 × PrimerSTAR Max Premix (TaKaRa), 10.5 μL ultrapure water, 1 μL of each primer (10 μM), and 1 μL of the cDNA template. An initial denaturation step at 98 °C for 2 min was followed by 35 cycles of 10 s at 98 °C, 15 s at 55 °C and 90 s at 72 °C, and a final extension step of 10 min at 72 °C. Purified PCR products were transferred to the vector pGEM-T Easy (Promega, Madison, WI) for sequencing (BGI, Beijing, China).

### Transcriptional profiling

Total RNA was extracted from (i) male and female antennae, maxillary palps, head cuticle (without antenna, maxillary palps, and proboscis), proboscis, legs, wings and ovipositors, and (ii) from the heads of 15-day-old virgin and mated females using TRIzol reagent (Invitrogen, Carlsbad, CA). Genomic DNA was eliminated with RNase-free DNase I (Promega) and first-strand cDNA was synthesized from 1 µg total RNA using the PrimeScript RT reagent kit (TaKaRa). Standard curves were used to evaluate primer efficiency (Supplementary Table 3) with fivefold serial dilutions of cDNA. Quantitative real-time PCR (qRT-PCR) was carried out using a CFX Connect Real-Time System (Bio-Rad, Hercules, CA) in a total reaction volume of 10 µL containing 5 μL SYBR Supermix (Novoprotein, Shanghai, China), 3.9 μL nuclease-free water, 0.5 μL cDNA (~200 ng/μL) and 0.3 μL of the forward and reverse primers (10 μM). We used *α-tubulin* (GenBank: GU269902) and *ribosomal protein S3* (GenBank: XM_011212815) as internal reference genes. Four biological replicates were prepared for each experiment. Relative expression levels were determined using the 2^−∆∆*Ct*^ method^43^, and data were analyzed using SPSS v20.0 (IBM).

### Two-electrode voltage clamp electrophysiological recordings

Verified PCR products representing candidate *B. dorsalis* OR genes and *BdorOrco* were transferred to vector pT7Ts for expression in oocytes. The plasmids were linearized for the synthesis of cRNAs using the mMESSAGE mMACHINE T7 Kit (Invitrogen, Lithuania). The purified cRNA was diluted to 2 µg/µL and ~60 ng cRNA was injected into *X. laevis* oocytes. The oocytes were pre-treated with 1.5 mg/mL collagenase I (GIBCO, Carlsbad, CA) in washing buffer (96 mM NaCl, 5 mM MgCl_2_, 2 mM KCl, 5 mM HEPES, pH 7.6) for 30–40 min at room temperature before injection. After incubation for 2 days at 18 °C in Ringer’s solution (96 mM NaCl, 5 mM MgCl_2_, 2 mM KCl, 5 mM HEPES, 0.8 mM CaCl_2_), the oocytes were exposed to different concentrations of 1-octen-3-ol diluted in Ringer’s solution from a 1 M stock in DMSO. Odorant-induced whole-cell inward currents were recorded from injected oocytes using a two-electrode voltage clamp and an OC-725C amplifier (Warner Instruments, Hamden, CT) at a holding potential of –80 mV. The signal was processed using a low-pass filter at 50 Hz and digitized at 1 kHz. Oocytes injected with nuclease-free water served as a negative control. Data were acquired using a Digidata 1550 A device (Warner Instruments, Hamden, CT) and analyzed using pCLAMP10.5 software (Axon Instruments Inc., Union City, CA).

### Calcium imaging assay

Verified PCR products representing candidate *B. dorsalis* OR genes and *BdorOrco* were transferred to vector pcDNA3.1(+) along with an mCherry tag that confers red fluorescence to confirm transfection. High-quality plasmid DNA was prepared using the Qiagen plasmid MIDIprep kit (QIAgen, Düsseldorf, Germany). The *B. dorsalis* OR and *BdorOrco* plasmids were co-transfected into HEK 293 cell using *Trans*IT-LT1 transfection reagent (Mirus Bio LLC, Japan) in 96-well plates. The fluorescent dye Fluo-4 AM (Invitrogen) was prepared as a 1 mM stock in DMSO and diluted to 2.5 μM in Hanks’ balanced salt solution (HBSS, Invitrogen, Lithuania) to serve as a calcium indicator. The cell culture medium was removed 24–30 h after transfection and cells were rinsed three times with HBSS before adding Fluo 4-AM and incubating the cells for 1 h in the dark. After three rinses in HBSS, 99 μL of fresh HBSS was added to each well before testing in the dark with 1 μL of diluted 1-octen-3-ol. Fluorescent images were acquired on a laser scanning confocal microscope (Zeiss, Germany). Fluo 4-AM was excited at 488 nm and mCherry at 555 nm. The relative change in fluorescence (ΔF/F0) was used to represent the change in Ca^2+^, where F0 is the baseline fluorescence and ΔF is the difference between the peak fluorescence induced by 1-octen-3-ol stimulation and the baseline. The healthy and successfully transfected cells (red when excited at 555 nm) were used for analysis. The final concentration of 10^−4^ M was initially used to screen corresponding ORs, and then to determine the response of screened ORs to stimulation with different concentrations of 1-octen-3-ol. Each concentration of 1-octen-3-ol was tested in triplicate. Concentration–response curves were prepared using GraphPad Prism v8.0 (GraphPad Software).

### Genome editing

The exon sequences of *BdorOR7a-6* and *BdorOR13a* were predicted using the high-quality *B. dorsalis* genome assembly. Each gRNA sequence was 20 nucleotides in length plus NGG as the protospacer adjacent motif (PAM). The potential for off-target mutations was evaluated by using CasOT to screen the *B. dorsalis* genome sequence. Each gRNA was synthesized using the GeneArt Precision gRNA Synthesis Kit (Invitrogen) and purified using the GeneArt gRNA Clean-up Kit (Invitrogen). Embryos were microinjected as previously described^20^. Purified gRNA and Cas9 protein from the GeneArt Platinum Cas9 Nuclease Kit (Invitrogen) were mixed and diluted to final concentrations of 600 and 500 ng/µL, respectively. Fresh eggs (laid within 20 min) were collected and exposed to 1% sodium hypochlorite for 90 s to soften the chorion. The eggs were fixed on glass slides and injected with the mix of gRNA and Cas9 protein at the posterior pole using an IM-300 device (Narishige, Tokyo, Japan) and needles prepared using a Model P-97 micropipette puller (Sutter Instrument Co, Novato, CA). Eggs were injected with nuclease-free water as a negative control. Injection was completed within 2 h. The injected embryos were cultured in a 27 °C incubator and mortality was recorded during subsequent development.

G_0_ mutants were screened as previously described^20^. G_0_ adult survivors were individually backcrossed to WT flies (single pair) to collect G_1_ offspring. Genomic DNA was extracted from G_0_ individuals after oviposition using the DNeasy Blood & Tissue Kit (Qiagen). The region surrounding each gRNA target was amplified by PCR using the extracted DNA as a template, the specific primers listed in Supplementary Table 2, and 2 × Taq PCR MasterMix (Biomed, Beijing, China). PCR products were analyzed by capillary electrophoresis using the QIAxcel DNA High Resolution Kit (Qiagen). PCR products differing from the WT alleles were purified and transferred to the vector pGEM-T Easy for sequencing. To confirm the mutation was inherited, genomic DNA was also extracted from one mesothoracic leg of G_1_ flies using InstaGene Matrix (Bio-Rad, Hercules, CA) and was analyzed as above. To avoid potential off-target mutations, heterozygous G_1_ mutants were backcrossed to WT flies more than 10 generations before self-crossing to generate homozygous mutant flies.

### Electroantennogram (EAG) recording

The antennal responses of 15-day-old *B. dorsalis* adults to 1-octen-3-ol were determined by EAG recording (Syntech, the Netherlands) as previously reported^20^. Briefly, antennae were fixed to two electrodes using Spectra 360 electrode gel (Parker, Fairfield, NJ, USA). The signal response was amplified using an IDAC4 device and collected using EAG-2000 software (Syntech). Before each experiment, 1-octen-3-ol and other three volatiles (ethyl tiglate, ethyl acetate, ethyl butyrate) were diluted to 10%, 1% and 0.1% (v/v) with MO to serve as the electrophysiological stimulus, and MO was used as a negative control. A constant air flow (100 mL/min) was produced using a controller (Syntech) to stimulate the antenna. We then placed 10 µL of each dilution or MO onto a piece of filter paper (5 × 1 cm), and the negative control (MO) was applied before and after the diluted odorants to calibrate the response signal. The EAG responses at each concentration were recorded for 15–20 antennae, and each concentration was recorded twice. Each test lasted 1 s, and the interval between tests was 30 s. EAG response data from WT and mutant flies for the diluted odorants were analyzed using Student’s *t* test with SPSS v20.0.

### Molecular docking and site-directed mutagenesis

The three dimensional-structures of BdorOR7a-6 and BdorOR13a were modeled using AlphaFold 2.0^44^. The quality and rationality of each protein structure was evaluated online using a PROCHECK Ramachandran plot in SAVES 6.0 (https://saves.mbi.ucla.edu/). AutoDock Vina 1.1.2 was used for docking analysis, and the receptor protein structure and ligand molecular structure were pre-treated using AutoDock 4.2.6. The docking parameters were set according to the protein structure and active sites, and the optimal docking model was selected based on affinity (kcal/mol). Docking models were imported into Pymol and Discovery Studio 2016 Client for analysis and image processing. Based on the molecular docking data, three residues (Asn86 in OR7a-6, Asp320, and Lys323 in OR13a) were replaced with alanine by site-directed mutagenesis^45^ using the primers listed in Supplementary Table 2. Calcium imaging assays and molecular docking of mutated proteins were then carried out as described above.

### Statistics reproducibility

All of the olfactory preference assays, oviposition bioassays, expression profiles analysis, EAG recording assays were analyzed using Student’s *t*-test (*p < 0.05, **p < 0.01, ***p < 0.001) with SPSS v20.0 (IBM). The figures were generated using GraphPad v8.0 (GraphPad Software). Sample size of each assay was stated in the manuscript.

### Reporting summary

Further information on research design is available in the Nature Portfolio Reporting Summary linked to this article.

## Supplementary information

Supplementary Information

Description of Additional Supplementary Files

Supplementary Moive 1

Supplementary Moive 2

Supplementary Moive 3

Supplementary Moive 4

Supplementary Moive 5

Supplementary Moive 6

Supplementary Data 1

Reporting Summary

## Data Availability

The final chromosome assembly genome data and the transcriptome data for maxillary palp and other tissues were submitted to CNGBdb under assembly accession number CNP0003192, CNP0003333, and CNP0003334, respectively. The deep transcriptome data for antenna was deposited in the National Center for Biotechnology Information Sequence Read Archive (SRA) under accession numbers SRR9026238.
