# Supplementary material for: Two odorant receptors regulate 1-octen-3-ol induced oviposition behavior in the oriental fruit fly
Source: Commun Biol. 2023 Feb 15;6:176. doi: 10.1038/s42003-023-04551-5 (PMC9932091; doi:10.1038/s42003-023-04551-5)
Supplement: Supplementary file 1 — Supplementary Information [file 42003_2023_4551_MOESM1_ESM.pdf]

Supplementary Figure 1. Phylogenetic tree of odorant receptors (ORs) identified in *B. dorsalis*. The maximum likelihood tree was constructed using iqtree with the best-fit model LG+F+R7. The branches in red are ORs from *B. dorsalis* whereas the branches in black are ORs from *D. melanogaster*.

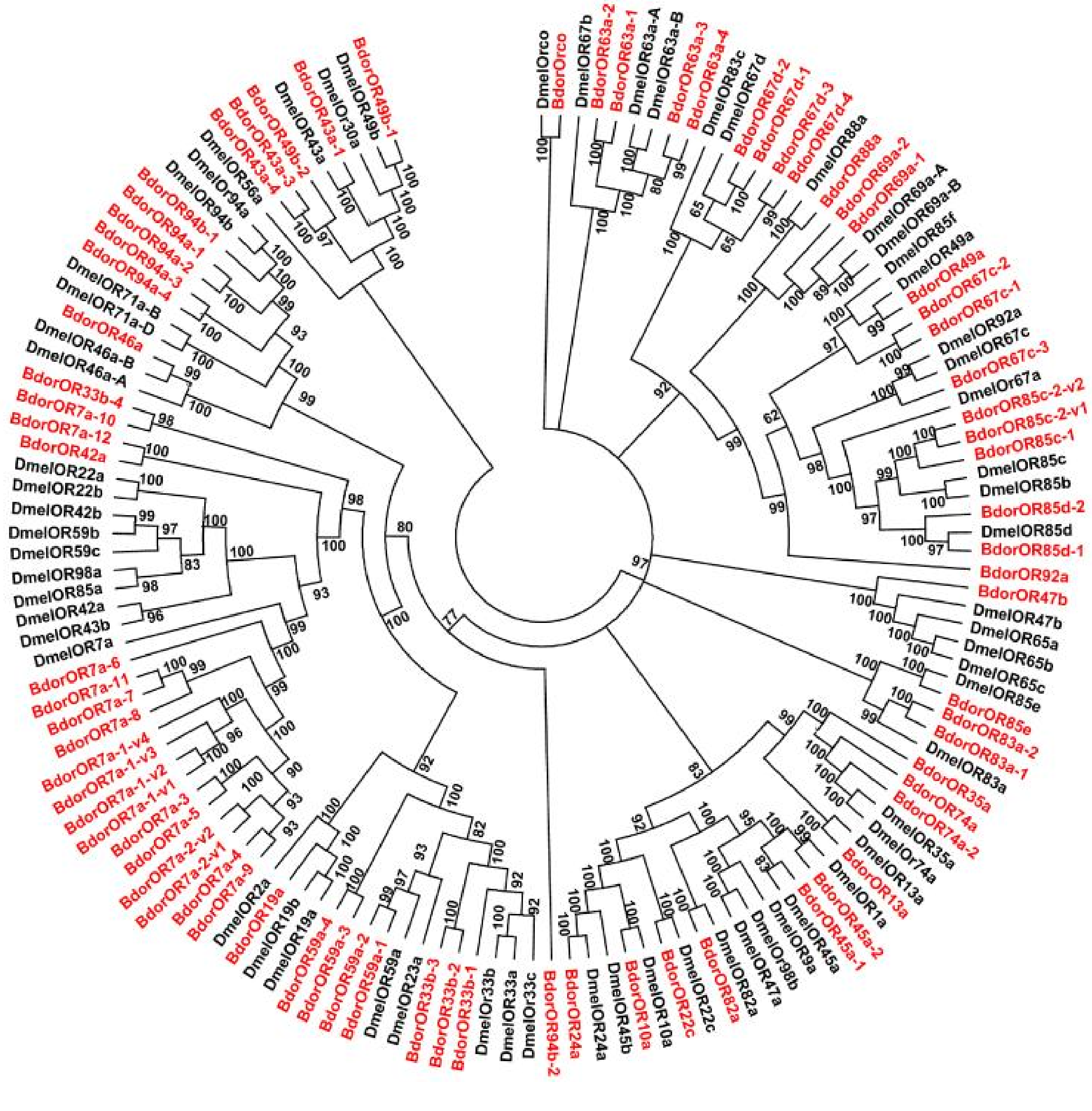

Supplementary Figure 2. The expression profiles of odorant receptors (ORs) in different *B. dorsalis* body segments. Each body segment included seven parts: antenna, maxillary palps, head cuticle, proboscis, legs, wings, ovipositors. These tissues were prepared separately from males and females. The heat map was generated using morpheus (https://software.broadinstitute.org/morpheus/). Each line represents a tissue, and each row represents a gene. The color scheme uses the minimum and maximum values in each line to establish a gradient of color intensity, with red representing higher and blue representing lower relative expression levels.

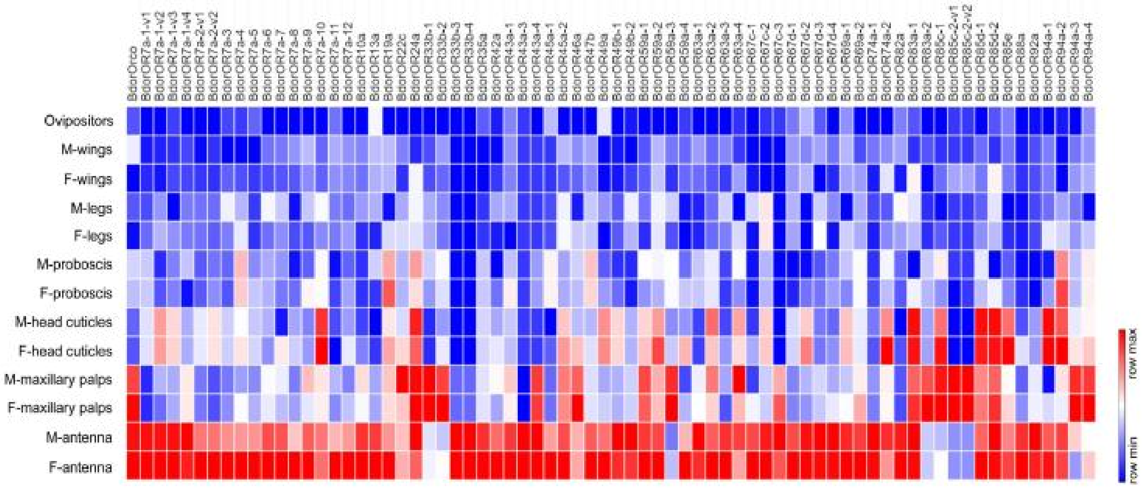

Supplementary Figure 3. The fold change in expression level of *B. dorsalis* odorant receptor (OR) genes when comparing virgin and mated flies. The red columns represent upregulated genes and the blue columns represent downregulated genes. The asterisks indicate statistically significant differences in the expression level of *B. dorsalis* OR genes between mated and virgin females based on Student's *t*-test (*$p < 0.05$; **$p < 0.01$).

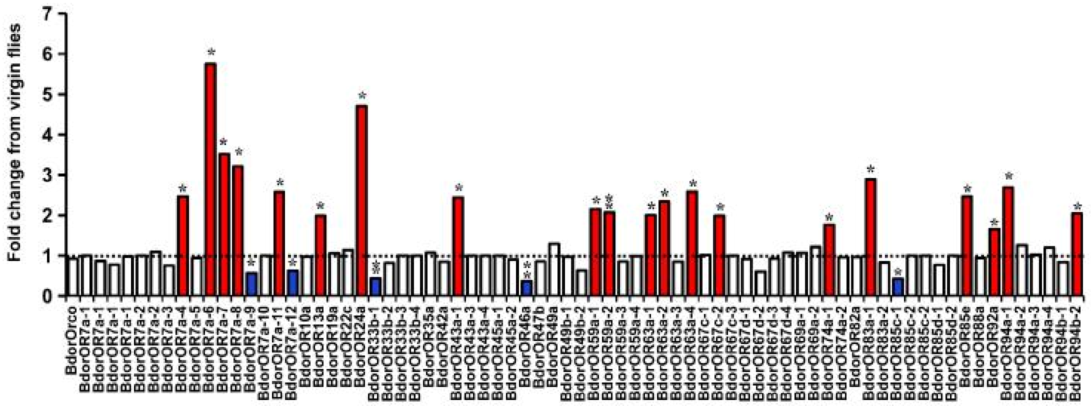

Supplementary Figure 4. Evaluation of BdorOR7a-6 and BdorOR13a using PROCHECK Ramachandran plots. A, B and L represent the residues in the most favored regions, whereas a, b, l and p represent residues in additional allowed regions, and ~a, ~b, ~l and ~p represent residues in generously allowed regions. (a) The evaluation of BdorOR7a-6 structure. (b) The evaluation of BdorOR13a structure.

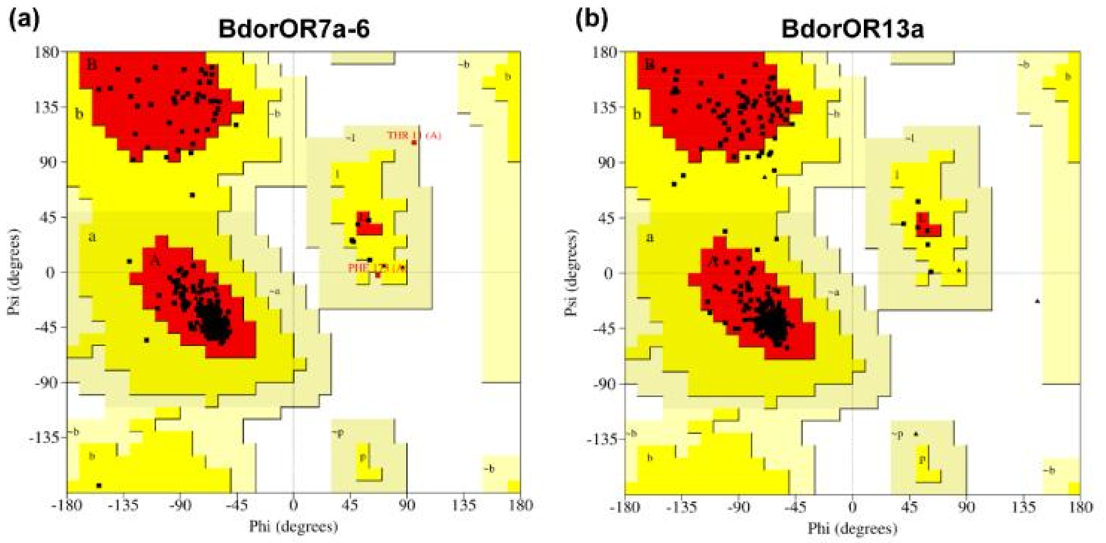

Supplementary Figure 5. Molecular docking of BdorOR7a-6 and BdorOR7a-6 Asn86Ala mutant to 1-octen-3-ol. (a) Residue Asn86 of BdorOR7a-6 forms hydrogen bond with 1-octen-3-ol. (b) Residue Asn86Ala of BdorOR7a-6 mutant could not form hydrogen bond with 1-octen-3-ol. (c) The conformation of BdorOR7a-6, and the region in red is residue Asn86. (d) The conformation of BdorOR7a-6 Asn86Ala mutant, and the region in blue is residue Asn86Ala.

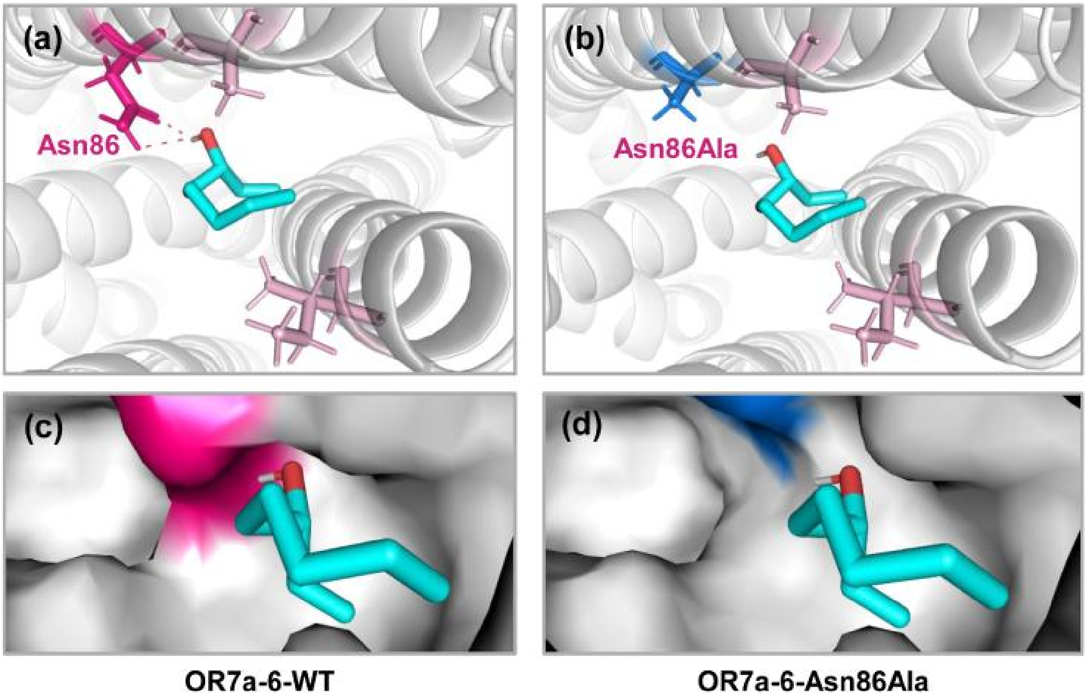

Supplementary Figure 6. Molecular docking of BdorOR13a and BdorOR13a Asp320Ala and Lys323Ala mutants to 1-octen-3-ol. (a) Residues Asp320 and Lys323 of BdorOR13a form hydrogen bond with 1-octen-3-ol. (b) Residue Asp320Ala of BdorOR13a mutant could not form hydrogen bond with 1-octen-3-ol. (c) Residue Lys323Ala of BdorOR13a mutant could not form hydrogen bond with 1-octen-3-ol. (d) The conformation of BdorOR13a, and the regions in red are residues Asp320 and Lys323. (e) The conformation of BdorOR13a Asp320Ala mutant. The region in red is residue Lys323, and the region in blue is residue Asp320Ala. (f) The conformation of BdorOR13a Lys323Ala mutant. The region in red is residue Asp320, and the region in blue is residue Lys323Ala.

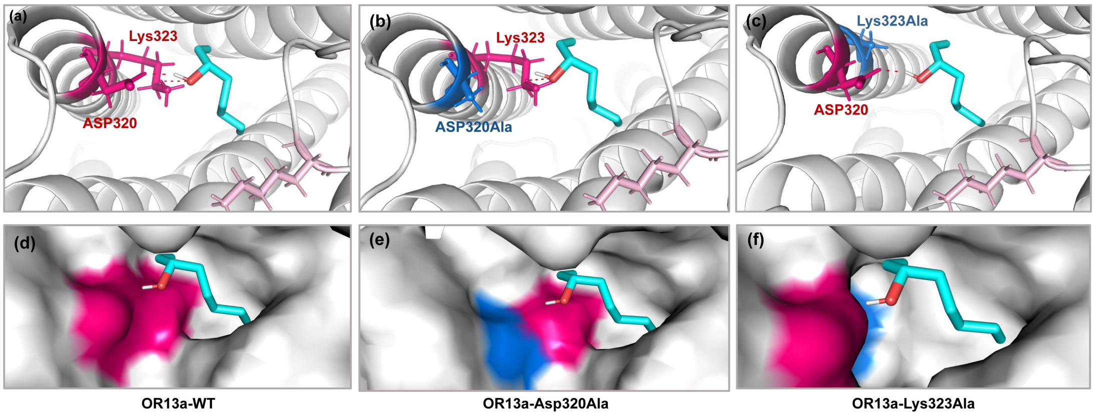

Supplementary Table 1. *Bactrocera dorsalis* odorant receptors.

Gene name	Length (AA)	BLAST best hit (Accession number; Name; Species)	Reference (Accession number or reference number)
BdorOrco	473	MT474521.1; ORco; *Bactrocera dorsalis*	Ref.1:KP743711.1; Ref.2: ADK97803.1
BdorOR7a-1-v1	391	MT474522.1; OR7a.1; *Bactrocera dorsalis*	Ref.2: XP_019845111.1
BdorOR7a-1-v2	386	MT474522.1; OR7a.1; *Bactrocera dorsalis*	Ref.2: XP_019845111.1
BdorOR7a-1-v3	391	MT474636.1; OR7a.1; *Bactrocera dorsalis*	Ref.2: XP_019845111.1
BdorOR7a-1-v4	392	MT474636.1; OR7a.1; *Bactrocera dorsalis*	Ref.2: XP_019845111.1
BdorOR7a-2-v1	401	MT474523.1; OR7a.2; *Bactrocera dorsalis*	Ref.2: XP_018798519.1
BdorOR7a-2-v2	400	MT474637.1; OR7a.2; *Bactrocera dorsalis*	Ref.2: XP_018798519.1
BdorOR7a-3	398	KP743714.1; OR7a-3; *Bactrocera dorsalis*	Ref.2: AKI29030.1
BdorOR7a-4	400	MT474639.1; OR7a-4; *Bactrocera dorsalis*	Ref.1: KP743721.1； Ref.2: XP_019846037.1
BdorOR7a-5	400	KP743716.1; OR7a-5; *Bactrocera dorsalis*	Ref.1:KP743716; Ref.2: XP_019845111
BdorOR7a-6	394	MT474527.1; OR7a-6; *Bactrocera dorsalis*	Ref.2: XP_011210512.1
BdorOR7a-7	394	MT474642.1; OR7a-7; *Bactrocera dorsalis*	Ref.1: KP743715.1; Ref.2: XP_011198720.1
BdorOR7a-8	394	MT474529.1; OR7a.8; *Bactrocera dorsalis*	Ref.1: KP743712.1; Ref.2: XP_019847175.1

BdorOR7a-9	399	MT474524.1; OR7a.3; *Bactrocera dorsalis*	Ref.2: XP_011208901.1
BdorOR7a-10	385	XM_029551853.1; OR7a-like; *Bactrocera dorsalis*	-
BdorOR7a-11	392	XM_011200916; OR7a; *Bactrocera dorsalis*	-
BdorOR7a-12	414	XM_011211139.3; OR7a-like; *Bactrocera dorsalis*	-
BdorOR10a	400	MT474644.1; OR10a; *Bactrocera dorsalis*	Ref.2: XP_018791769.1
BdorOR13a	441	MT474531.1; OR13a.1; *Bactrocera dorsalis*	Ref.1:MT474531; Ref.2: AKI29033
BdorOR19a	393	MT474533.1; OR19a; *Bactrocera dorsalis*	Ref.2: XP_011198390.1
BdorOR22c	400	XM_011211808.1; OR22c; *Bactrocera dorsalis*	-
BdorOR24a	422	XM_011201220.1; OR24a; *Bactrocera dorsalis*	Ref.2: XP_011199522.1
BdorOR33b-1	389	XM_011209638.2; OR33b-like; *Bactrocera dorsalis*	-
BdorOR33b-2	377	XM_029551535.1; OR33b-like; *Bactrocera dorsalis*	-
BdorOR33b-3	376	XM_029551534.1; OR33b-like; *Bactrocera dorsalis*	
BdorOR33b-4	388	XM_011210598.1; OR33b-like; *Bactrocera dorsalis*	
BdorOR35a	417	XM_019988878.2; OR35a; *Bactrocera dorsalis*	Ref.1: KP743718; Ref.2: XP_019844437
BdorOR42a	465	XP_029407913.1; OR7a; *Bactrocera dorsalis*	Ref.1: KP743713.1; Ref.2: XP_019847361
BdorOR43a-1	378	MT474535.1; OR43a-1; *Bactrocera dorsalis*	Ref.1: KP743719； Ref.2: AKI29035

BdorOR43a-3	381	MT474651.1; OR43a-3; *Bactrocera dorsalis*	Ref.1: XP_019847596.1
BdorOR43a-4	375	MT474538.1; OR43a-4; *Bactrocera dorsalis*	Ref.1: KP743720; Ref.2: AKI29036
BdorOR45a-1	390	MT474539.1; R45a; *Bactrocera dorsalis*	Ref.2: XP_011212447.2
BdorOR45a-2	430	XM_040096630.1; OR45a-like; *Bactrocera tryoni*	-
BdorOR46a	388	XM_029549903.1; OR46a; *Bactrocera dorsalis*	-
BdorOR47b	449	MT474655; OR47b; *Bactrocera dorsalis*	Ref.2: XP_019847427
BdorOR49a	394	MT474656; OR49a; *Bactrocera dorsalis*	Ref.2: XP_011212431
BdorOR49b-1	371	KP743723.1; OR49b-1; *Bactrocera dorsalis*	Ref.1: KP743723.1; Ref.2: XP_019845516.1
BdorOR49b-2	400	KP743724.1; OR49b-2; *Bactrocera dorsalis*	Ref.2: XP_019847679
BdorOR59a-1	388	MT474543.1; OR59a.1; *Bactrocera dorsalis*	Ref.1:KP743725; Ref.2: AKI29041
BdorOR59a-2	648	XM_011203958.1; OR59a-like; *Bactrocera dorsalis*	-
BdorOR59a-3	381	XM_011201169; OR59a-like; *Bactrocera dorsalis*	-
BdorOR59a-4	387	XM_040108763.1; OR59a-like; *Bactrocera tryoni*	-
BdorOR63a-1	415	KP743726; OR63a-1; *Bactrocera dorsalis*	Ref.1:AKI29042; Ref.2: KP743726.1
BdorOR63a-2	417	KP743727; OR63a-2; *Bactrocera dorsalis*	Ref.1: KP743727; Ref.2: AKI29043
BdorOR63a-3	414	MT474545; OR63a-1; *Bactrocera dorsalis*	Ref.2: XP_018783180
BdorOR63a-4	414	XM_029549744.1; OR63a-like; *Bactrocera dorsalis*	Ref.2: XP_018783180.1

BdorOR67c-1	405	KP743728.1; OR67c; *Bactrocera dorsalis*	Ref.1: KP743728.1; Ref.2: XP_011200400.1
BdorOR67c-2	405	XM_029549367.1; OR67c-like; *Bactrocera dorsalis*	Ref.1: KP743728.1; Ref.2: XP_011200400.1
BdorOR67c-3	403	XM_011200850.3; OR67c-like; *Bactrocera dorsalis*	-
BdorOR67d-1	386	MT474549.1; OR67d.1; *Bactrocera dorsalis*	Ref.2: XP_017473047.1
BdorOR67d-2	387	MT474550.1; OR67d.2; *Bactrocera dorsalis*	Ref.2: XP_017473047.1
BdorOR67d-3	388	MT474551.1; OR67d-3; *Bactrocera dorsalis*	Ref.2: XP_011203704
BdorOR67d-4	388	MT474666.1; OR67d.4; *Bactrocera dorsalis*	Ref.1: KP743729.1; Ref.2: XP_011203703.1
BdorOR69a-1	414	XM_011211067.3; OR69a; *Bactrocera dorsalis*	Ref.1: KP743730.1； Ref.2: AKI29046.1
BdorOR69a-2	423	MT474554.1; OR69a.2; *Bactrocera dorsalis*	Ref.2: XP_011191113.1
BdorOR74a-1	427	MT474669.1; OR74a; *Bactrocera dorsalis*	Ref.1:KP743731.1; Ref.2: XP_011201924.2
BdorOR74a-2	402	XM_014236173.2; OR74a; Bactrocera oleae	-
BdorOR82a	399	MT474556.1; OR82a; *Bactrocera dorsalis*	Ref.2: XP_011208732
BdorOR83a-1	473	XM_011205570.2; OR83a; *Bactrocera dorsalis*	-
BdorOR83a-2	470	XM_011205479.2; OR83a-like; *Bactrocera dorsalis*	-
BdorOR85c-1	402	XM_011211273.3; OR85c-like isoform X1; *Bactrocera dorsalis*	-
BdorOR85c-2-v1	412	XM_011211274.2; OR85c-like; *Bactrocera dorsalis*	-

BdorOR85c-2-v2	412	XM_011211274.2; OR85c-like; *Bactrocera dorsalis*	-
BdorOR85d-1	423	XM_018946194.1; OR85d; *Bactrocera latifrons*	-
BdorOR85d-2	420	XM_011211276.2; OR85d; *Bactrocera dorsalis*	Ref.2: XP_018801738.1
BdorOR85e	450	XM_018945342.1; OR85e; *Bactrocera dorsalis*	-
BdorOR88a	420	MT474671; OR88a; *Bactrocera dorsalis*	Ref.1:KP743732; Ref.2: AKI29043
BdorOR92a	384	MT474672.1; OR92a; *Bactrocera dorsalis*	Ref.2: XP_011208819.1
BdorOR94a-1	389	XM_011203454.1; OR94a-like; *Bactrocera dorsalis*	-
BdorOR94a-2	387	MT474673.1; OR94a; *Bactrocera dorsalis*	-
BdorOR94a-3	383	XM_040097304.1; OR94a-like; *Bactrocera tryoni*	-
BdorOR94a-4	383	XM_014239851.2; OR94a-like; *Bactrocera oleae*	-
BdorOR94b-1	396	MT474559.1; OR94b.1; *Bactrocera dorsalis*	Ref.1:KP743733.1; Ref.2: XP_019847876.1
BdorOR94b-2	402	MT474675; OR94b.2; *Bactrocera dorsalis*	Ref.2: XP_018801531

Ref.1: Wu et al., 2015; Ref.2: Miyazaki et al., 2018

Supplementary Table 2. Primer sequences used in this study for purposes other than qPCR.

Experiment types	Primer name	Nucleotide sequences (5′→3′)
Voltage-clamp recording and calcium imaging	OR7a-6-F	ATAAGAATGCGGCCGCGCCACCATGTCGAAGATATTACTTGTTCG
	OR7a-6-R	ATAAGAATGCGGCCGCTTAATTTTCCGTATCAGTGCC
	OR13a-F	ATAAGAATGCGGCCGCGCCACCCTTTAAGATGTTATTCAATCCG
	OR13a-R	ATAAGAATGCGGCCGCCCACTGCCACTGCTTAGTT
	Orco-F	ATAAGAATGCGGCCGCGCCACCATGCAGCCCAGCAAATATG
	Orco-R	ATAAGAATGCGGCCGCTACTTCAATTGCACCAGCA
gRNA synthesis	OR7a-6-F	TAATACGACTCACTATAGCGCGCGGGTGCGCTGACTG
	OR7a-6-R	TTCTAGCTCTAAAACCAGTCAGCGCACCCGCGCGC
	OR13a-F	TAATACGACTCACTATAGTACTGTATTCTGCCTTTGG
	OR13a-R	TTCTAGCTCTAAAACCCAAAGGCAGAATACAGTAC
Genotyping	OR7a-6-F	GGAGTACTGGACGATTGTGTT
	OR7a-6-R	CGTCATTTCTGTCACTCTTTTC
	OR13a-F	CAACACAGCTCCTCACACC
	OR13a-R	GAATGCATTCGACGTCTG

Site-directed mutagenesis	ASN86-OR7a-6-FL	CTGGCCGCCATACAAGCGGCCGTGCAAGTATC
	ASN86-OR7a-6-RL	GCACGGCCGCTTGTATGGCGGCCAGTAATTGGC
	ASN86-OR7a-6-FS	CAAGTATCGGGCATCCCT
	ASN86-OR7a-6-RS	GTAATTGGCTGTTGGTTAGC
	ASP320-OR13a-FL	CATGTTCATTGGTGCGTACGTAAAGTTTTTGG
	ASP320-OR13a-RL	ACTTTACGTACGCACCAATGAACATGTTTTGAC
	ASP320-OR13a-FS	CGTAAAGTTTTTGGTTTAC
	ASP320-OR13a-RS	TGAACATGTTTTGACCCGTC
	LYS323-OR13a-FL	GGTGATTACGTAGCGTTTTTGGTTTAC
	LYS323-OR13a-RL	CCAAAAACGCTACGTAATCACCAATGAAC
	LYS323-OR13a-FS	GGTTTACATTCTCTCATCGCTC
	LYS323-OR13a-RS	CACCAATGAACATGTTTTGACC

Supplementary Table 3.. Primer sequences used in this study for qPCR.

Gene name	Primer (5′→3′)	Gene name	Primer (5′→3′)
BdorOrco-F	ACATTGTTGGCATATCAGGC	*BdorOR49b-2-F*	ATCCTTGATGTCGCGTTTCC
BdorOrco-R	CTCGGAGCCATCATACCAGT	*BdorOR49b-2-R*	GCTTCCTCGTCGGTCATTTC
BdorOR7a-1-v1-F	TATCAGCCCCTACGACACTG	*BdorOR59a-1-F*	GGAGAGTCCTGCTGCCAATG
BdorOR7a-1-v1-R	CTGCTCGTCGTTAAAGTCGG	*BdorOR59a-1-R*	ATAGTAATGCGGCCACAGGT
BdorOR7a-1-v2-F	TTTGCAAGCATCGCTTAATG	*BdorOR59a-2-F*	TAACTTGTTGCCTGCTGTCG
BdorOR7a-1-v2-R	GGCTTCTGCAAGTCTTTTCG	*BdorOR59a-2-R*	GAATGAACATCGGCCAGGAC
BdorOR7a-1-v3-F	GTGCCTTACGCACCCATATT	*BdorOR59a-3-F*	GCAGCATAACCAGGACTTCG
BdorOR7a-1-v3-R	TTGCAGAAAAACCGTCAGTG	*BdorOR59a-3-R*	TTAGCGCGCCGAACATAAAA
BdorOR7a-1-v4-F	TGGATTCTTTCACGTTGCGC	*BdorOR59a-4-F*	TGCAAATTCTGGTTACCGCC
BdorOR7a-1-v4-R	TCAGTTGCCAGATTGTTCGC	*BdorOR59a-4-R*	ACTCCAACTGCATGCAAGTG
BdorOR7a-2-v1-F	GCTGTCGGCTTTGTTTTCTC	*BdorOR63a-1-F*	CTTTAGGAACCTGCGTCTGC
BdorOR7a-2-v1-R	TGAGTGAGGTTTGCATCAGC	*BdorOR63a-1-R*	CGTAGTACGGTGAGGCCATT
BdorOR7a-2-v2-F	GCAAATCTCTAGGGCTGTCG	*BdorOR63a-2-F*	CTCGCCTTATTATGCAGTGG
BdorOR7a-2-v2-R	CGATGCCTGATAGCAAGTGA	*BdorOR63a-2-R*	ACTTGGAGTGAGGTGGATCG
BdorOR7a-3-F	ACTATCTATGCGGCCCTTAT	*BdorOR63a-3-F*	GCAGTTGAGGCAAGAGATCG

BdorOR7a-3-R	CGTTTATCCAAAGCCAGCCA	*BdorOR63a-3-R*	CCGGAACAGGCAGAAAGAAC
BdorOR7a-4-F	GCAGCACCGCATATCTTCAA	*BdorOR63a-4-F*	AATGGCCAGCACCTAACAAC
BdorOR7a-4-R	CACCCAAATGACTAACGCGT	*BdorOR63a-4-R*	AAGGTGGCCAAATTCATCAG
BdorOR7a-5-F	ACTTGCGCATTTAGGAGTCG	*BdorOR67c-1-F*	TTTAAGAGCAGTGAACCCGC
BdorOR7a-5-R	TGGCTACCACCCAACTTGAT	*BdorOR67c-1-R*	AGATGTCGCTAACTCGTCGT
BdorOR7a-6-F	TATGAGGAGCTGTTGGGATG	*BdorOR67c-2-F*	GACATGAAAGTGCTCGGACC
BdorOR7a-6-R	GCAACAGTTGGCAGGAGAAA	*BdorOR67c-2-R*	AATGACGATCACAGCCTCCG
BdorOR7a-7-F	CGTATTGCTCCAGACTTCGC	*BdorOR67c-3-F*	TACAAATTGCCACAACGCGA
BdorOR7a-7-R	GGCACCAATTTGAGAGCGAA	*BdorOR67c-3-R*	GTGAGCCAGTAGACCCAAGT
BdorOR7a-8-F	TGAGCAGGATCGGCGTTTTC	*BdorOR67d-1-F*	ATAGTATTCTGGGCACGCGT
BdorOR7a-8-R	TTCCTGACCAATGCCCATCT	*BdorOR67d-1-R*	ACTGCACGACCATCATAGCT
BdorOR7a-9-F	TGGTATCCTTTTCTCGACTG	*BdorOR67d-2-F*	TCGTGTGCTTTCTCTGGCTA
BdorOR7a-9-R	CGACCAATGCGTTCCCTTAG	*BdorOR67d-2-R*	TATATTTCGTGGTGCCTGCG
BdorOR7a-10-F	CTGCTCTATTTGGGCGTCAC	*BdorOR67d-3-F*	ATCTTGTGAACGTGTGTGGC
BdorOR7a-10-R	CGCAGCGAGAAACCAATGTA	*BdorOR67d-3-R*	GTGTCGACATCAATGCCAGG
BdorOR7a-11-F	GCCTTGTCATTGGGTCGTTT	*BdorOR67d-4-F*	CCAATGAAGTCTCCGGCATG
BdorOR7a-11-R	GAGCGATGCCAATCCACAAA	*BdorOR67d-4-R*	ACAATTGCCGCACCGTAAAT

BdorOR7a-12-F	CCTCTTAGGGCTCTACGACG	*BdorOR69a-1-F*	CAACGGAACCCTTCACTTGG
BdorOR7a-12-R	ACAACGACCGCCAGTATGTA	*BdorOR69a-1-R*	CCAGTTTCGGTTACCTGCAC
BdorOR10a-F	GTACTTTTCCACTGCGCGAT	*BdorOR69a-2-F*	GTACCGATTCTGCAGCTGTG
BdorOR10a-R	GTGTCCAAAGCGAGTTCCAG	*BdorOR69a-2-R*	CAGACATACTCCGCAATCGC
BdorOR13a-F	TCCTGCTCAAATCTTTCGGT	*BdorOR74a-1-v1-F*	TCGCCAAATCAATGCAGGAG
BdorOR13a-R	TGGTAATGAGGCTGGACTGG	*BdorOR74a-1-v1-R*	GGCGTCCAAAGATCAGCAAT
BdorOR19a-F	ATCGATTCGGCCAAGAAATG	*BdorOR74a-1-v2-F*	GTACGACCGCTTCTTTTGGG
BdorOR19a-R	AACCATACGCGATCCAGAAC	*BdorOR74a-1-v2-R*	CAGGAAACGCTTGAATGGCT
BdorOR22c-F	GCTGCTCTTATCTGGTTCGC	*BdorOR82a-F*	TGATTAGCATGATTTGGGCG
BdorOR22c-R	CGCAGAGGAAGAAACCATCG	*BdorOR82a-R*	CGCAGATTTGTGGTGAACGA
BdorOR24a-F	GTCGGGCATAACATTGGGAC	*BdorOR83a-1-F*	TTTGCTGTTATGCTGGCGTT
BdorOR24a-R	ATTCAATGACCACTGTGCCG	*BdorOR83a-1-R*	AGAAAGCACTCGTACAGCCA
BdorOR33b-1-F	CAGACGAGCGCAACTACTTC	*BdorOR83a-2-F*	CAGCGCCTACTTCTTGTCAC
BdorOR33b-1-R	TCGTGTGCCAAGTTTTGTGT	*BdorOR83a-2-R*	TCTCCTCATTTGGCATCCGT
BdorOR33b-2-F	TGGCGTCTTCTCGGTATCAC	*BdorOR85c-1-F*	GCTGGCTATCATTCGGCTTC
BdorOR33b-2-R	ATGCATGCCACACTGATGAC	*BdorOR85c-1-R*	CGGTATGACTCGCTCTCCTT
BdorOR33b-3-F	GCATGCTTTCGGGTCATATT	*BdorOR85c-2-v1-F*	ATATTGAACGCGTGCTACCC

BdorOR33b-3-R	AGAGATTGTCGGCGAAGAAA	*BdorOR85c-2-v1-R*	GCCATACACTTCGGCAAACT
BdorOR33b-4-F	GACAACTACGCTGGACACCA	*BdorOR85c-2-v2-F*	TTACCACCATTGACGTGTGC
BdorOR33b-4-R	CACGGCAACACTGATATTGG	*BdorOR85c-2-v2-R*	AGACCGCCAGCTTCATTAGT
BdorOR35a-F	CAAGGCTCATTTCACCCACC	*BdorOR85d-1-F*	TGCGCGAAATTTCACCTTCA
BdorOR35a-R	CCAACAAATCCTCCCAACGG	*BdorOR85d-1-R*	CAACAGTGAAGGCATGCGTA
BdorOR42a-F	GCAGCTTCTTCATACTGGCC	*BdorOR85d-2-F*	ACATGCGCTCTATCCCCAAA
BdorOR42a-R	AAACATGGGACGTTGTGAGC	*BdorOR85d-2-R*	CCGCCAGCCAAACTTCATAG
BdorOR43a-1-F	CCCGTCTGCCTAATGAATGC	*BdorOR85e-F*	GGCTGTGGACTGGGCTATAT
BdorOR43a-1-R	ACCAATTCTTCCATCAGCGC	*BdorOR85e-R*	AGCCAATAGACGTTGCCAAC
BdorOR43a-3-F	GTGAGACGGGCATGAATTTT	*BdorOR88a-F*	GCTATTTCTGGCAGTGGTGG
BdorOR43a-3-R	TGACGTCCTGCAGCATAAAG	*BdorOR88a-R*	TTGCTGCAGATGTTCGCTAC
BdorOR43a-4-F	CTTATGTTCGGCGAATTGGT	*BdorOR92a-F*	CAGCTGATGTTGCACTTGGA
BdorOR43a-4-R	AGAGCGCAACGTGAAAGAAT	*BdorOR92a-R*	TGATGAAGAGGCCAAGCTGA
BdorOR45a-1-F	TCCAGTCGAAATTGGTCACA	*BdorOR94a-1-F*	TGGTCGACTTCTTTACGGCT
BdorOR45a-1-R	TAAATCCACCGTGTCCACAA	*BdorOR94a-1-R*	CAATCGAATGGTGGCGCATA
BdorOR45a-2-F	TCTGGGTGACATATCTGGGC	*BdorOR94a-2-F*	GGAGCTTGCATTATTGGCCA
BdorOR45a-2-R	AACCCCGTTAAATTGGTGGC	*BdorOR94a-2-R*	GCGATTGTCACCAGCGTTAT

BdorOR46a-F	ATCCACGACGAAGATCTGCT	*BdorOR94a-3-F*	AACTCGGCATTCTGTGCTTT
BdorOR46a-R	TCGCCAGGCTCCGATATAAG	*BdorOR94a-3-R*	GCTGATTACCCGGAAACAAA
BdorOR47b-F	GGTTCAGCCTCCCCATACTT	*BdorOR94a-4-F*	CTCGGTGTTCATGGCTTTGT
BdorOR47b-R	TGTCAACATTTCCCATGCGG	*BdorOR94a-4-R*	GCTCCATCCTCATGCCAATC
BdorOR49a-F	GCATGGATAACTCAAAGGCAGA	*BdorOR94b-1-F*	AAGTGGCCAACGATCCCATA
BdorOR49a-R	CGAAACCAAAGCCCTCCAAT	*BdorOR94b-1-R*	CATCGGCTGGTGTCATCATG
BdorOR49b-1-F	TGATTACATTGCGCGCGTAA	*BdorOR94b-2-F*	CGAAGTGGCTTTTGTGGTGA
BdorOR49b-1-R	CAGCTCGTTCGCATACCAAT	*BdorOR94b-2-R*	AGGGGAATATTTGACCGGCA

Supplementary Data 1 (Data sets file). The amino acids of odorant receptors.
