# Supplementary material for: Two odorant receptors regulate 1-octen-3-ol induced oviposition behavior in the oriental fruit fly
Source: Commun Biol. 2023 Feb 15;6:176. doi: 10.1038/s42003-023-04551-5 (PMC9932091; doi:10.1038/s42003-023-04551-5)
Supplement: Supplementary file 2 — Description of Additional Supplementary Files [file 42003_2023_4551_MOESM2_ESM.pdf]

Description of Additional Supplementary Files

Supplementary Movie 1: The oviposition preference of wild-type gravid females when offered a choice between mango fruit and mineral oil (negative control). Video has been accelerated 30 times for more complete presentation.

Supplementary Movie 2: The oviposition preference of wild-type gravid females when offered a choice between 1-octen-3-ol and mineral oil (negative control). Video has been accelerated 30 times for more complete presentation.

Supplementary Movie 3: The oviposition preference of wild-type gravid females when offered a choice between 1-octen-3-ol and mango fruit. Video has been accelerated 30 times for more complete presentation.

Supplementary Movie 4: The oviposition preference of wild-type gravid females when offered a choice between mango flesh plus 1- octen-3-ol and mango flesh plus mineral oil. Video has been accelerated 30 times for more complete presentation.

Supplementary Movie 5: The oviposition preference of BdorOR7a-6–/– mutant females when offered a choice between 1-octen-3-ol and mineral oil (negative control). Video has been accelerated 30 times for more complete presentation.

Supplementary Movie 6: The oviposition preference of BdorOR13a–/– mutant females when offered a choice between 1-octen-3-ol and mineral oil (negative control). Video has been accelerated 30 times for more complete presentation.

Supplementary Data 1 : The amino acids of odorant receptors.
