# Supplementary material for: Two odorant receptors regulate 1-octen-3-ol induced oviposition behavior in the oriental fruit fly
Source: Commun Biol. 2023 Feb 15;6:176. doi: 10.1038/s42003-023-04551-5 (PMC9932091; doi:10.1038/s42003-023-04551-5)
Supplement: Supplementary file 10 — Reporting Summary [file 42003_2023_4551_MOESM10_ESM.pdf]

nature portfolio

Corresponding author(s): Jin-jun Wang

Last updated by author(s): Dec 28, 2022

Reporting Summary

Nature Portfolio wishes to improve the reproducibility of the work that we publish. This form provides structure for consistency and transparency in reporting. For further information on Nature Portfolio policies, see our Editorial Policies and the Editorial Policy Checklist.

Statistics

For all statistical analyses, confirm that the following items are present in the figure legend, table legend, main text, or Methods section.

n/a	Confirmed	
☐	☒	The exact sample size (n) for each experimental group/condition, given as a discrete number and unit of measurement
☐	☒	A statement on whether measurements were taken from distinct samples or whether the same sample was measured repeatedly
☐	☒	The statistical test(s) used AND whether they are one- or two-sided<br>*Only common tests should be described solely by name; describe more complex techniques in the Methods section.*
☐	☒	A description of all covariates tested
☐	☒	A description of any assumptions or corrections, such as tests of normality and adjustment for multiple comparisons
☐	☒	A full description of the statistical parameters including central tendency (e.g. means) or other basic estimates (e.g. regression coefficient) AND variation (e.g. standard deviation) or associated estimates of uncertainty (e.g. confidence intervals)
☐	☒	For null hypothesis testing, the test statistic (e.g. F, t, r) with confidence intervals, effect sizes, degrees of freedom and P value noted<br>*Give P values as exact values whenever suitable.*
☒	☐	For Bayesian analysis, information on the choice of priors and Markov chain Monte Carlo settings
☒	☐	For hierarchical and complex designs, identification of the appropriate level for tests and full reporting of outcomes
☒	☐	Estimates of effect sizes (e.g. Cohen's d, Pearson's r), indicating how they were calculated

Our web collection on statistics for biologists contains articles on many of the points above.

Software and code

Policy information about availability of computer code

Data collection	The EAG responses were recorded by GcEAD v1.2 software (Syntech, Netherlands).<br>The electrophysiological responses of oocytes were recorded by Digidata Digidata 1550A device (Warner Instruments, Hamden, CT) and analyzed using pCLAMP10.5 software (Axon Instruments Inc., Union City, CA).<br>Fluorescent intensity of HEK cells was recorded by software ZEN 2 (Zesis, Germany) .<br>The protein structure of odorant receptors were obtained from AlphaFold 2.0.
Data analysis	Video was analyzed using EthoVision XT software (16 version, Noldus Information Technology).<br>Concentration–response curves were analyzed using GraphPad Prism v8.0.<br>The quality and rationality of each protein structure was evaluated online using a PROCHECK Ramachandran plot in SAVES 6.0 (https://saves.mbi.ucla.edu/).<br>AutoDock Vina 1.1.2 was used for docking analysis, and the receptor protein structure and ligand molecular structure were pre-treated using AutoDock 4.2.6.<br>Data were analyzed using SPSS v20.0 (SPSS Inc.,Chicago, IL, USA).

For manuscripts utilizing custom algorithms or software that are central to the research but not yet described in published literature, software must be made available to editors and reviewers. We strongly encourage code deposition in a community repository (e.g. GitHub). See the Nature Portfolio guidelines for submitting code & software for further information.

Data

Policy information about availability of data

All manuscripts must include a data availability statement. This statement should provide the following information, where applicable:

- Accession codes, unique identifiers, or web links for publicly available datasets
- A description of any restrictions on data availability
- For clinical datasets or third party data, please ensure that the statement adheres to our policy

All data generated or analyzed during this study are included in this article (and its supplementary information files).

Human research participants

Policy information about studies involving human research participants and Sex and Gender in Research.

Reporting on sex and gender	*Use the terms sex (biological attribute) and gender (shaped by social and cultural circumstances) carefully in order to avoid confusing both terms. Indicate if findings apply to only one sex or gender; describe whether sex and gender were considered in study design whether sex and/or gender was determined based on self-reporting or assigned and methods used. Provide in the source data disaggregated sex and gender data where this information has been collected, and consent has been obtained for sharing of individual-level data; provide overall numbers in this Reporting Summary. Please state if this information has not been collected. Report sex- and gender-based analyses where performed, justify reasons for lack of sex- and gender-based analysis.*
Population characteristics	*Describe the covariate-relevant population characteristics of the human research participants (e.g. age, genotypic information, past and current diagnosis and treatment categories). If you filled out the behavioural & social sciences study design questions and have nothing to add here, write "See above."*
Recruitment	*Describe how participants were recruited. Outline any potential self-selection bias or other biases that may be present and how these are likely to impact results.*
Ethics oversight	*Identify the organization(s) that approved the study protocol.*

Note that full information on the approval of the study protocol must also be provided in the manuscript.

Field-specific reporting

Please select the one below that is the best fit for your research. If you are not sure, read the appropriate sections before making your selection.

☒ Life sciences ☐ Behavioural & social sciences ☐ Ecological, evolutionary & environmental sciences

For a reference copy of the document with all sections, see nature.com/documents/nr-reporting-summary-flat.pdf

Life sciences study design

All studies must disclose on these points even when the disclosure is negative.

Sample size	For behavioral assays, each group contained 30 flies and each experiment contained 7-15 replications. The sample sizes were sufficient.<br>For electrophysiological recordings, sample size was determined based on our previous study (Xu et al., 2022).<br>For voltage clamp recording, sample size was determined based previous study (Hou et al., 2020).<br>For genome editting assay, sample size was determined based on our previous study (Xu et al., 2022).
Data exclusions	No data were excluded from analysis.
Replication	All experiments were replicated at least four times, and all attempts at replication were successful.
Randomization	All samples/organisms/participants were allocated into experimental groups randomly.
Blinding	The investigations were blinded to group allocation during data collection and/or analysis.

Reporting for specific materials, systems and methods

We require information from authors about some types of materials, experimental systems and methods used in many studies. Here, indicate whether each material, system or method listed is relevant to your study. If you are not sure if a list item applies to your research, read the appropriate section before selecting a response.

Materials & experimental systems

n/a	Involved in the study
☒	☐ Antibodies
☐	☒ Eukaryotic cell lines
☒	☐ Palaeontology and archaeology
☐	☒ Animals and other organisms
☒	☐ Clinical data
☒	☐ Dual use research of concern

Methods

n/a	Involved in the study
☒	☐ ChIP-seq
☒	☐ Flow cytometry
☒	☐ MRI-based neuroimaging

Eukaryotic cell lines

Policy information about cell lines and Sex and Gender in Research

Cell line source(s)	HEK293 cell (Human embryonic kidney cell 293), a cell line derived from human embryonic kidney cells. We purchased from ThermoFisher Scientific (Waltham, MA).
Authentication	HEK cells were authenticated by company from which we purchased (ThermoFisher Scientific, Waltham, MA).
Mycoplasma contamination	The cell lines were not tested for Mycoplasma contamination.
Commonly misidentified lines (See ICLAC register)	No commonly misidentified lines.

Animals and other research organisms

Policy information about studies involving animals; ARRIVE guidelines recommended for reporting animal research, and Sex and Gender in Research

Laboratory animals	The oriental fruit flies (Bactrocera dorsalis, males and females, eggs, larvae, pupae, adults), and frogs (Xenepus laevis, females adults older than two years) used in the experiments were maintained in the Key Laboratory of Entomology and Pest Control Engineering in Chongqing, China.
Wild animals	Study did not involve wild animals.
Reporting on sex	This study focused on the oviposition behavior mediated by female oriental fruit flies. Therefore, the behavioral assays were designed for female flies. In addition, the female Xenepus laevis was used to provide Xenepus oocytes for voltage clamp recording.
Field-collected samples	The study did not involve samples collected from the field for laboratory work.
Ethics oversight	All experiments were conducted under the license of the Animal Experimental Committee of South West university.

Note that full information on the approval of the study protocol must also be provided in the manuscript.
